# Designing DNA nanostar hydrogels for sequence-specific degradation and antibody release

**DOI:** 10.1039/d6sm00563b

**Published:** 2026-08-05

**Authors:** Giorgia Palombo, Christine A. Merrick, Jennifer Harnett, Susan Rosser, Davide Michieletto, Yair Augusto Gutiérrez Fosado

**Affiliations:** a School of Physics and Astronomy, University of Edinburgh, Peter Guthrie Tait Road Edinburgh EH9 3FD UK davide.michieletto@ed.ac.uk yair.fosado@ed.ac.uk; b Centre for Engineering Biology, School of Biological Sciences, University of Edinburgh Edinburgh UK; c MRC Human Genetics Unit, Institute of Genetics and Cancer, University of Edinburgh UK; d International Institute for Sustainability with Knotted Chiral Meta Matter (WPI-SKCM^2^), Hiroshima University Higashi-Hiroshima Hiroshima 739-8526 Japan

## Abstract

DNA nanostar (DNAns) hydrogels are promising materials for *in vivo* applications, including tissue regeneration and drug and antibody delivery. However, a systematic and quantitative understanding of the design principles controlling their degradation is lacking. Here, we investigate hydrogels made of three-armed DNAns with varying flexible joints, arm lengths, and mesh sizes and use restriction enzymes (RE) to cut the DNAns structures while monitoring the gel's degradation. We discover that (i) removing flexible joints, (ii) increasing arm length, or (iii) relocating the RE site along a DNA linker markedly accelerates hydrogel degradation. In contrast, non-specific endonucleases, *e.g.* DNaseI, quickly degrade DNAns hydrogels regardless of design. Importantly, the release of antibodies from DNAns hydrogels can be modulated by the action of sequence-specific enzymes, confirming that design-dependent susceptibility to sequence-specific enzymatic degradation can be leveraged for responsive drug-delivery systems. These findings provide new design principles for engineering DNAns hydrogels with tailored material properties, sequence-specific enzymatic susceptibility, and controlled cargo release.

## Abstract 2

DNA nanostar hydrogels functionalised with antibodies undergo sequence-specific degradation by restriction enzymes, triggering the release of the antibodies.

## Graphical abstract

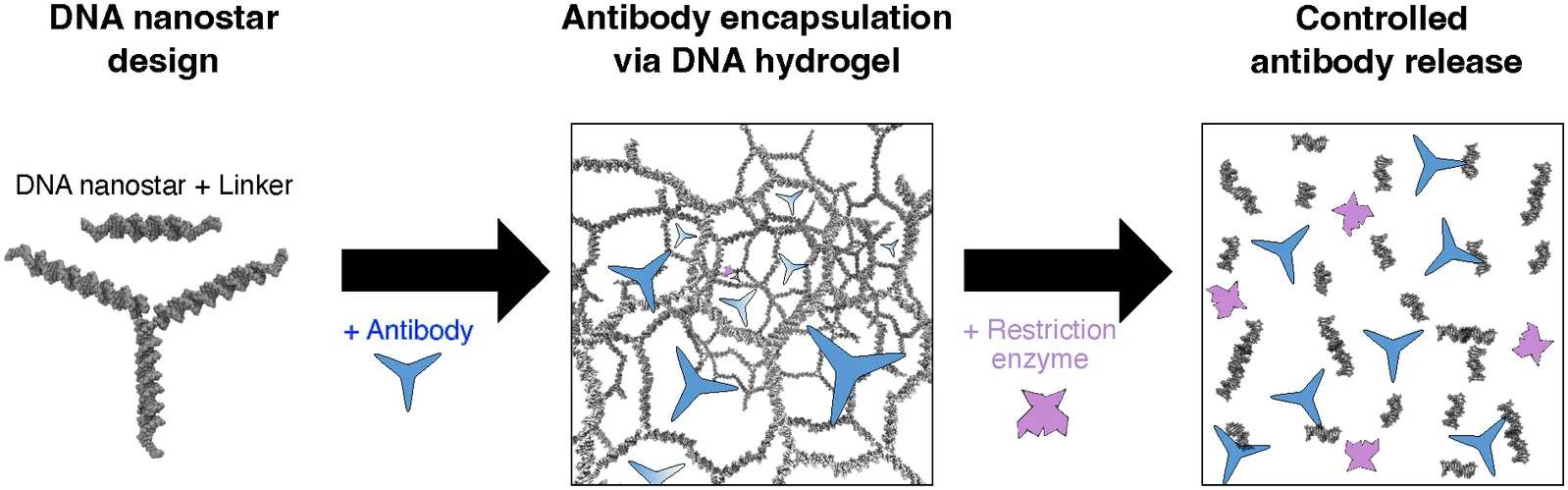

## 1. Introduction

**Fig. 1:**
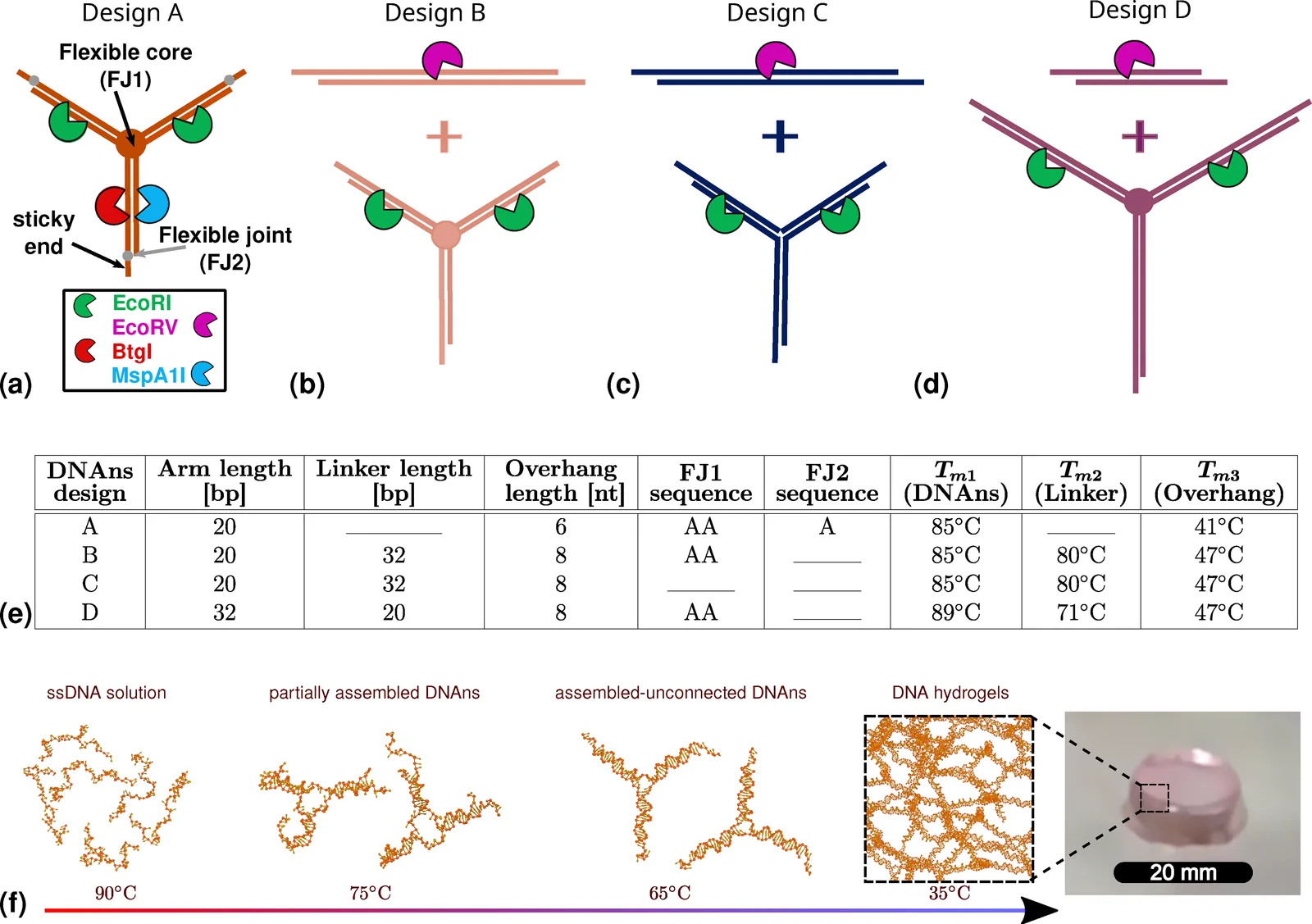
DNA nanostar designs. (a)–(d) Schematic representations of the four DNAns designs used in this study. Each design self-assembles into a three-armed DNA nanostar containing recognition sequences for the restriction enzymes EcoRI-HF (two arms, labeled in green), BtgI, and MspA1I (remaining arm, labeled in red and cyan, respectively). For designs B-D an EcoRV-HF site (labelled in magenta) is located on the linker. (e) Summary table of structural parameters for each design. Columns two to four list arm, linker, and sticky-end lengths (in base-pairs (bp) and nucleotides (nt)). Columns five and six indicate the number of unpaired adenines (A) forming flexible joints at the core (FJ1) and before the sticky ends (FJ2). The final three columns report the melting temperatures determined with NUPACK^22^ at [NaCl] = 150 mM, [Mg^2+^] = 10 mM and [DNA] = 250 µM. (f) Schematic of the annealing protocol used for DNAns and hydrogel assembly (shown for design A, without linker).

Deoxyribonucleic acid (DNA) nanotechnology has rapidly progressed from fundamental research to applied technology.^1^ Its unmatched molecular specificity and programmability make DNA an ideal material for constructing self-assembling, responsive, and highly tunable nanostructures.^1,2^ Among these, DNA nanostars (DNAns) have emerged as versatile building blocks, serving both as model systems for probing the properties of molecular liquids^3–7^ and as functional materials^8^ for tissue regeneration,^9^ biosensing,^10^ synthetic cells,^11–13^ and drug delivery.^14,15^ DNAns are formed from short single-stranded DNA (ssDNA) oligomers designed to hybridize into multi-armed, double-helical motifs emanating from a central junction^16^ (Fig. 1). The arms terminate in short self-complementary ssDNA overhangs, or “sticky ends”, which mediate the assembly of adjacent DNAns into higher-order networks. By programming arm number (or valence), arm length, junction flexibility, and overhang length, DNAns can be precisely tuned to control the mechanical properties, connectivity, and responsiveness of the resulting material.^3,6,17–20^ At high concentrations, DNAns percolate into soft, hydrophilic networks known as DNA hydrogels,^3,4,6,16–18,21^ whose programmable structure offers unique opportunities for controlled biomolecule encapsulation, triggered release, and responsive degradation, key features for the next-generation of biomedical applications.

The mechanical properties of DNAns hydrogels can be tuned *via* chemical and physical methods,^14^ as well as by modulating the specific structure and connectivity of the DNAns building blocks.^17,19,23,24^ In addition, nature provides a diverse toolkit of enzymes capable of manipulating DNA with high precision and modifying the ensuing material properties in dense and entangled solutions of long DNA molecules.^25–28^ However, the precise rheological control of DNAns hydrogels through DNA-altering enzymes has so far been largely unexplored despite being a promising route to programmable degradation *in vivo*.

To address this gap, here we investigate the enzymatic degradation of DNAns hydrogels using restriction enzymes (REs) and systematically quantify the degradation of, and antibody release from, DNAns hydrogels. Notably, while the enzymatic degradation of DNAns liquid droplets has been comprehensively examined in recent studies^29,30^ (see also SI, Fig. S3), the degradation of the “gel” regime is unexplored.

By combining confocal microscopy, time-resolved microrheology, and gel electrophoresis, we investigated the enzymatic degradation of DNAns hydrogels made with different DNAns designs and across different phases.

One of the key findings of our work is that, under the experimental conditions tested, non-specific endonucleases such as DNaseI readily induce network cleavage, whereas site-specific REs show limited impact on gel integrity. By systematically testing different structural designs, we found that sequence-specific enzymatic degradation of DNAns hydrogels is governed primarily by the accessibility of restriction sites within the DNA nanostar architecture rather than by the hydrogel network architecture. Importantly, this accessibility can be modulated through nanostar design, while different designs also provide programmable control over the material properties of the resulting hydrogels.

Finally, we demonstrate that a degradable DNA nanostar hydrogel can be used for the encapsulation and enzyme-triggered release of a humanized monoclonal IgG, a model antibody currently used in clinical trials for cancer treatment. Together, these findings establish molecular design as a powerful strategy for engineering DNA hydrogels with tailored material properties, design-dependent susceptibility to sequence-specific enzymatic degradation, and controlled cargo release, advancing the development of stimuli-responsive scaffolds for tissue regeneration, 3D cell culture, and drug delivery.

## 2. Results and discussion

To systematically explore how nanostar structural features influence enzymatic degradation, we designed four DNAns variants, labeled A–D (see Fig. 1). In design A, the sticky ends are self-complementary, allowing direct hybridization between adjacent nanostars. In contrast, designs B–D feature non-self-complementary sticky ends that hybridize only with the overhangs of double-stranded DNA linkers, which act as bridges connecting neighboring nanostars. This architectural difference enables control over network connectivity and pore size, while variations in core and joint flexibility determine the molecular dynamics and accessibility of the restriction sites. Detailed sequences, annealing protocols for each design and sample preparations are provided in the Methods section and SI (Sections SI, SII and SIII).

In the following sections, we study the degradation properties of DNAns gels through a combination of gel electrophoresis and time-resolved microrheology. This dual approach allows us to visually identify digested DNA fragments and correlate the digestion with the rheological properties of the gel.

### 2.1. Resistance to site-specific endonucleases in the linker-free DNA nanostar hydrogel

**Fig. 2:**
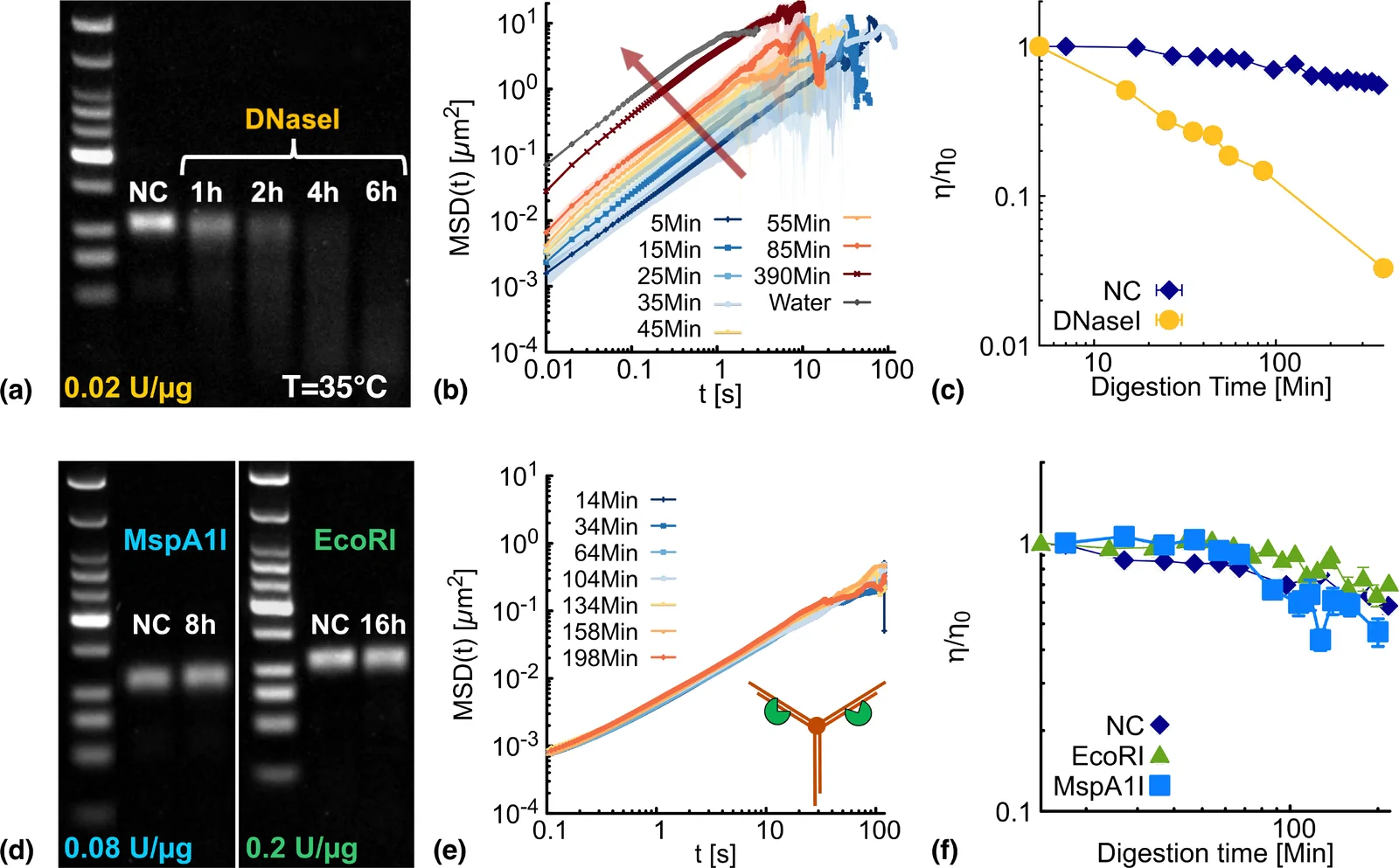
DNA hydrogels formed by design A nanostars display RE resistance. (a) Agarose gel electrophoresis (native conditions) of DNA fragments from DNAns hydrogels (250 µM, corresponding to ∼120 µg µL^−1^) treated with DNaseI for different incubation times (1, 2, 4 and 6 hours) at 35 °C. “NC” is negative control (no addition of RE). Ladder is NEB low molecular weight. (b) Time-resolved microrheology shows that the gel progressively loses viscoelasticity. (c) Normalized viscosity decreases monotonically with time. (d) Agarose gel electrophoresis (native conditions) of DNAns hydrogels (at 250 µM) treated with the sequence-specific restriction enzymes MspA1I (8 h incubation) and EcoRI (16 h incubation) shows no detectable degradation. (e) Microrheology confirms no significant change in material properties with respect to control. (f) Normalized viscosity as a function of time display no significant loss of integrity. In panels (c) and (f) the viscosity at digestion time t is normalized by the initial value (*η*_0_), measured at the earliest available time point.

We first examined the enzymatic digestion of hydrogels formed by design A DNAns upon treatment with the non-specific and highly processive DNaseI (with an enzyme(U)-to-DNA(µg) ratio of 0.02 U µg^−1^, *i.e.* 2 U of enzyme in 120 µg of DNAns). As shown in Fig. 2(a), the negative control (no enzyme) displays a single band, corresponding to intact design A nanostars (running around 100 bp). Upon addition of DNaseI, the bands intensity gradually decreases and eventually disappears after approximately 4 hours, indicating complete digestion of the DNAns (see SI, Section SVI for DNaseI digestion of designs B–D). This observation is confirmed by microrheology measurements: Fig. 2(b) reports the mean-squared displacement (MSD) of *a* = 200 nm tracer beads, MSD(*t*, *t*_d_) = 〈[***r***(*t* + *t*_0_; *t*_d_) − ***r***(*t*_0_; *t*_d_)]^2^〉, where the average is performed over tracers, positions in the sample and initial times *t*_0_. The “digestion time” *t*_d_ ≫ *t*_0_ denotes the elapsed time since enzyme addition, while the lagtime *t* denotes the time elapsed between two positions. Typically, we record 2 minutes videos every 10 minutes over several hours, allowing us to extract sequential MSD curves (MSD(*t*, 5 min), MSD(*t*, 15 min),…) and monitor the temporal evolution of sample viscosity. As digestion proceeds, the MSD curves shift upward, reflecting a progressive decrease in viscosity, *i.e.* the beads diffuse faster in the hydrogel. The corresponding viscosity, *η*(*t*_d_) = *k*_B_*T*/(3π*D*_p_(*t*_d_)*a*), where 
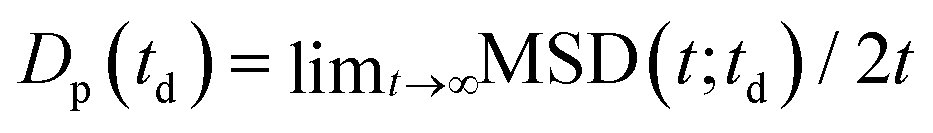
, is plotted as a function of digestion time in Fig. 2(c). In the negative control we observe a mild reduction in viscosity, which may be associated with the presence of glycerol in the enzyme buffer (see SI, Section SVI). In contrast, the DNaseI-treated sample displays a ∼33-fold reduction in viscosity within six hours.

We next performed analogous experiments using sequence-specific REs to test whether they could degrade design A hydrogel. We used MspA1I (0.08 U µg^−1^, 10 U enzyme in 120 µg DNA) and EcoRI (0.2 U µg^−1^, 20 U enzyme in 120 µg DNA). For comparison, in the phase-separation regime, DNAns droplets are completely digested by EcoRI at a much higher stoichiometry (20 U µg^−1^, 20 U enzyme and 1 µg DNA) within approximately 800 s (see SI, Fig. S3). Achieving comparable enzyme-to-DNA ratios in bulk hydrogels would require enzyme quantities that are impossible to attain at the much higher DNA concentrations used here. Even when using the highest feasible enzyme concentrations, a naive linear extrapolation predicts gel degradation on a timescale of ∼22 hours, roughly 100-fold longer than observed for the droplets. However, no measurable digestion was detected over these timescales. Surprisingly, both gel electrophoresis (Fig. 2(d)) and microrheology (Fig. 2(e and f)) show that the hydrogel remains intact over time. This lack of degradation occurred despite the use of enzyme-to-DNA mass ratios four times higher for MspA1I and ten times higher for EcoRI compared to the DNaseI experiments, in which complete digestion was observed. Indeed, the behaviour of samples treated with sequence-specific enzymes is indistinguishable from that of the negative controls (Fig. 2(f)).

We note that enzyme activity units are defined under assay-specific conditions (see SI Section SIII) and do not directly reflect cleavage efficiency across different DNA substrates. Therefore, all comparisons in this study are based on the observed activity under our experimental conditions. Within this framework, DNaseI is nonetheless intrinsically several orders of magnitude more processive than the other REs examined here.

The resistance of the linker-free DNA nanostar hydrogel to site-specific restriction enzyme degradation was unexpected, especially given previous observations of enzymatic degradation in DNAns droplets^29,30^ and qualitative visual effects on the stability of DNAns gels.^31^ Moreover, under the same experimental conditions, the hydrogels are readily degraded by the non-specific endonuclease DNaseI. We therefore sought to investigate whether the resistance to restriction enzymes originates from the network architecture or from the local accessibility of the restriction sites within the DNA nanostars. To examine the role of the network architecture, in the next section we investigate whether reducing the network connectivity facilitates enzymatic degradation.

### 2.2. Changes in network architecture do not affect the degradation efficiency of DNAns hydrogels

**Fig. 3:**
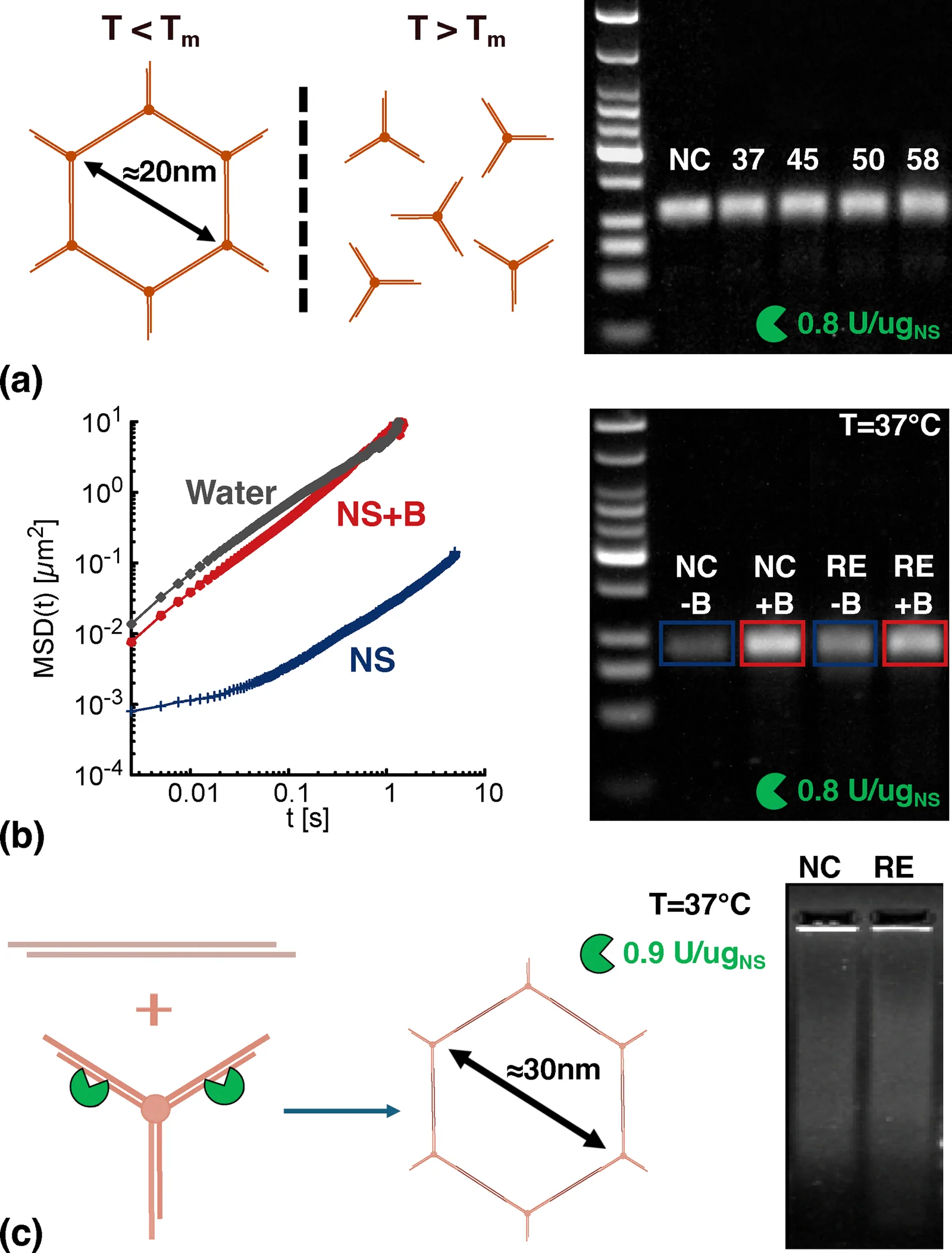
Hydrogel pore size does not affect EcoRI-mediated degradation. (a) Left: schematic of design A DNAns gels below and above the sticky-end melting temperature (*T*_m3_ = 41 °C). Right: agarose gel electrophoresis (native conditions) of design A hydrogels incubated with EcoRI for 16 h at 37–58 °C. (b) Left: MSD from microrheology for water (gray), design A hydrogel (blue), and hydrogel with ssDNA blockers (red). Right: Agarose gel electrophoresis (native conditions) of design A hydrogels with (+B) and without (−B) blocking strands. All samples were incubated for 16 h with EcoRI at 37 °C. Relevant comparisons are between the control (NC) and EcoRI-treated (RE) samples within each experimental condition (−B and +B), highlighted in blue and red, respectively. (c) Left: schematic of a design B nanostar hydrogel displaying larger pores. Right: agarose gel electrophoresis (native conditions) of design B hydrogels incubated for 16 h with EcoRI at 37 °C and showing no degradation with respect to negative control (NC).

To assess whether enzyme degradation efficiency depends on the network's structure, we conducted a series of experiments using different DNAns designs while maintaining a constant EcoRI-to-DNA ratio of approximately 0.8 U µg^−1^ (100 U EcoRI per ∼120 µg DNA), corresponding to the highest number of EcoRI units commercially available. We first investigated whether increasing temperature, and thereby modifying the network connectivity, affected EcoRI-mediated degradation of design A nanostar hydrogels. As the temperature approaches the sticky-end melting temperature (*T*_m3_ ≃ 41 °C), overhang bonds weaken, potentially enlarging the pore size. Above *T*_m3_, the network is expected to lose connectivity and transition towards a liquid-like state, which could facilitate enzyme accessibility. To test this, design A hydrogels were incubated with EcoRI for 16 h at fixed temperatures of 37 °C, 45 °C, 50 °C, and 58 °C. Gel electrophoresis revealed no detectable degradation at any temperature (Fig. 3(a)). This outcome is unexpected, as all tested temperatures lie below the EcoRI inactivation temperature (65 °C), with 37 °C corresponding to its optimal activity. These results indicate that reducing network connectivity alone does not enhance restriction enzyme efficiency in DNA hydrogel degradation. We note, however, that prolonged incubation at elevated temperatures may reduce the effective enzymatic activity. To address this limitation, we complemented these experiments with the blocker-strand approach described below, which probes the same hypothesis under the optimal EcoRI activity condition (37 °C).

We introduced oligonucleotides complementary to the sticky ends of design A nanostars in a 3 : 1 molar ratio. These short strands (6 nucleotides (nt) long) act as blockers, preventing DNAns to bind with each other and in turn disrupting network formation. Microrheology confirmed that DNAns containing blocking strands exhibited water-like viscosity and no elasticity (Fig. 3(b), left). Comparison of the gel electrophoresis results for the control (NC) and EcoRI-treated (RE) samples within each experimental condition (*i.e.*, in the absence and presence of blocker strands; Fig. 3(b), right) revealed no detectable EcoRI-mediated degradation after 16 h of incubation at 37 °C, irrespective of the presence of blocker strands.

Finally, we investigated the digestion of a DNAns design that includes a double stranded DNA linker between DNAns, hence enlarging the pore size of the mesh. More specifically, DNAns design B retains the same arm length, flexibility, and EcoRI site placement as design A, but it includes a dsDNA linker that extends the core-to-core contour length distance by ∼10 nm. This distance (comprising one nanostar arm, sticky end, the linker, and another arm) totals 88 bp, corresponding to ∼30 nm (assuming 0.34 nm per bp). Also, the longer sticky-ends (8 nt in design B *vs.* 6 nt in design A) form highly stable, larger structures that remain trapped in the well in gel electrophoresis (see negative control (NC) in Fig. 3(c)). Despite these modifications, no detectable digestion was observed after 21 hours of incubation at 37 °C at 0.9 U µg^−1^ (0.45 U µg^−1^ when accounting for both nanostars and linkers), with the DNAns structures remaining in the well, similar to the control.

In conclusion, these experiments collectively indicate that, under the conditions investigated, the pore size and fluidity of the DNAns network do not determine the efficiency of site-specific RE digestion in DNAns hydrogels. Although the enzymes can diffuse through the network, they still fail to locate and cleave their recognition sites effectively. These observations suggest that the limited digestion is more likely related to the local accessibility of the restriction sites within the individual DNA nanostar architecture than to the hydrogel pore structure. While the present experiments do not allow us to identify the underlying molecular mechanism, geometric and/or steric hindrance imposed by the nanostar architecture represents a plausible explanation, consistent with the enhanced resistance to enzymatic digestion previously reported for DNA tetrahedral nanostructures.^32^ Other factors, such as local changes in the ionic environment arising from counterion association with the dense DNA network, may also contribute to the observed reduction in enzymatic activity.

### 2.3. Relocating restriction site to the linker enhances enzymatic cleavage

To investigate whether the location of restriction sites affects cleavage efficiency, we compared two restriction enzymes: EcoRI, which upon cleavage produces additional sticky ends, and EcoRV, which generates blunt ends. Both enzymes locate their target sequences by sliding along the DNA, but differ in recognition and cleavage mechanisms:^33^ EcoRI relies on precise hydrogen bonding, whereas EcoRV bends the DNA by ∼50°, allowing cleavage *via* conformational changes. These differences are particularly relevant in crowded environments, such as DNAns hydrogels, where steric accessibility can limit enzymatic activity.

We designed two variants of design C DNAns: version (i) displays EcoRI sites on two nanostar arms and one EcoRV site on the linker (NS(I) + L(V)), and version (ii) obtained by swapping the EcoRI and EcoRV sites from the first version (NS(V) + L(I)). Compared to design B, design C lacks a flexible core. We hypothesise that if the reason for EcoRI failing to cleave DNAns design B was steric and/or geometric hindrance, then placing the restriction sites on the linker should minimizes these constraints and increase the likelihood of efficient cleavage.

**Fig. 4:**
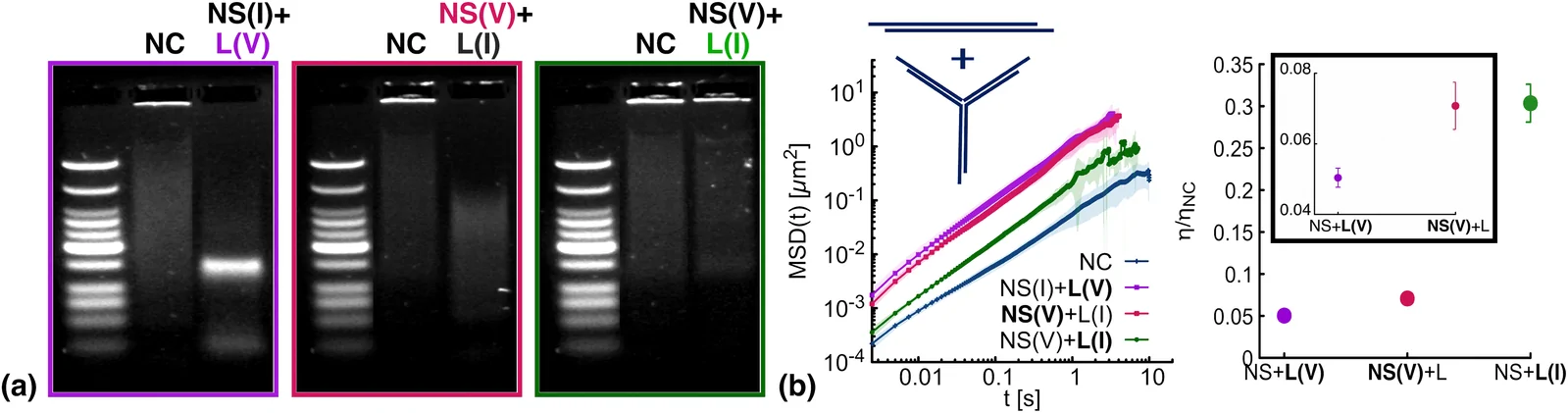
Effect of site location on DNAns hydrogel cleavage by EcoRI and EcoRV. (a) Agarose gel electrophoresis (native conditions) after 21 h digestion at 37 °C and 0.68 U µg^−1^. Left: EcoRV digestion of NS(I) + L(V) (site on linker, purple). Middle: EcoRV digestion of NS(V) + L(I) (site on two arms, magenta). Right: EcoRI digestion of NS(V) + L(I) (site on linker, green). NC: samples without enzyme. (b) Left: MSD from microrheology experiments for NC (blue) and digested samples (EcoRV: purple/magenta, EcoRI: green), showing increased mobility after digestion. Right: Normalized viscosity (*η*/*η*_NC_) for each case: EcoRV at linker, EcoRV on DNAns arms, EcoRI on linker.

Consistent with this hypothesis, gel electrophoresis and microrheology measurements (at 0.68 U µg^−1^, 100 U enzyme in 148 µg mass of linker and DNAns) reported in Fig. 4(a and b), show that EcoRV cleaves most efficiently when the recognition site is on the linker, while partial cleavage occurs for EcoRV at the arms. Furthermore, we observe minimal cleavage for EcoRI at the linker. The effect of EcoRI can best be seen in microrheology (Fig. 4(b)), where the NS(V) + L(I) sample displays lower viscosity than the control but not as low as when the DNAns design C is cut with EcoRV.

These results highlight the critical role of enzyme choice and restriction site placement for optimizing DNA hydrogel degradation. Relocating the restriction site to the linker (thus farther from the nanostar core) and using the blunt-end enzyme EcoRV (instead of EcoRI) enhances digestion efficiency.

### 2.4. DNAns can be designed to encode hydrogel viscoelasticity pre/post degradation

Having identified conditions under which DNAns hydrogels can undergo degradation by site-specific REs, we next study how intrinsic DNAns design features influence the mechanical response and degradation kinetics of the hydrogels. Specifically, we compared the behaviour of hydrogels made with DNAns designs B and D (where we swapped EcoRI and EcoRV recognition sites for each design in Fig. 1(b and d)) when digested by EcoRV (100 U) at 37 °C with an enzyme-to-DNA ratio of 0.9 U µg^−1^. Both designs have a flexible core but differ in arm length and linker length (see Fig. 1(e)). These architectural differences yield hydrogels with distinct initial viscoelastic properties and provide a means to assess how network geometry influences both baseline mechanics and enzymatic softening.

**Fig. 5:**
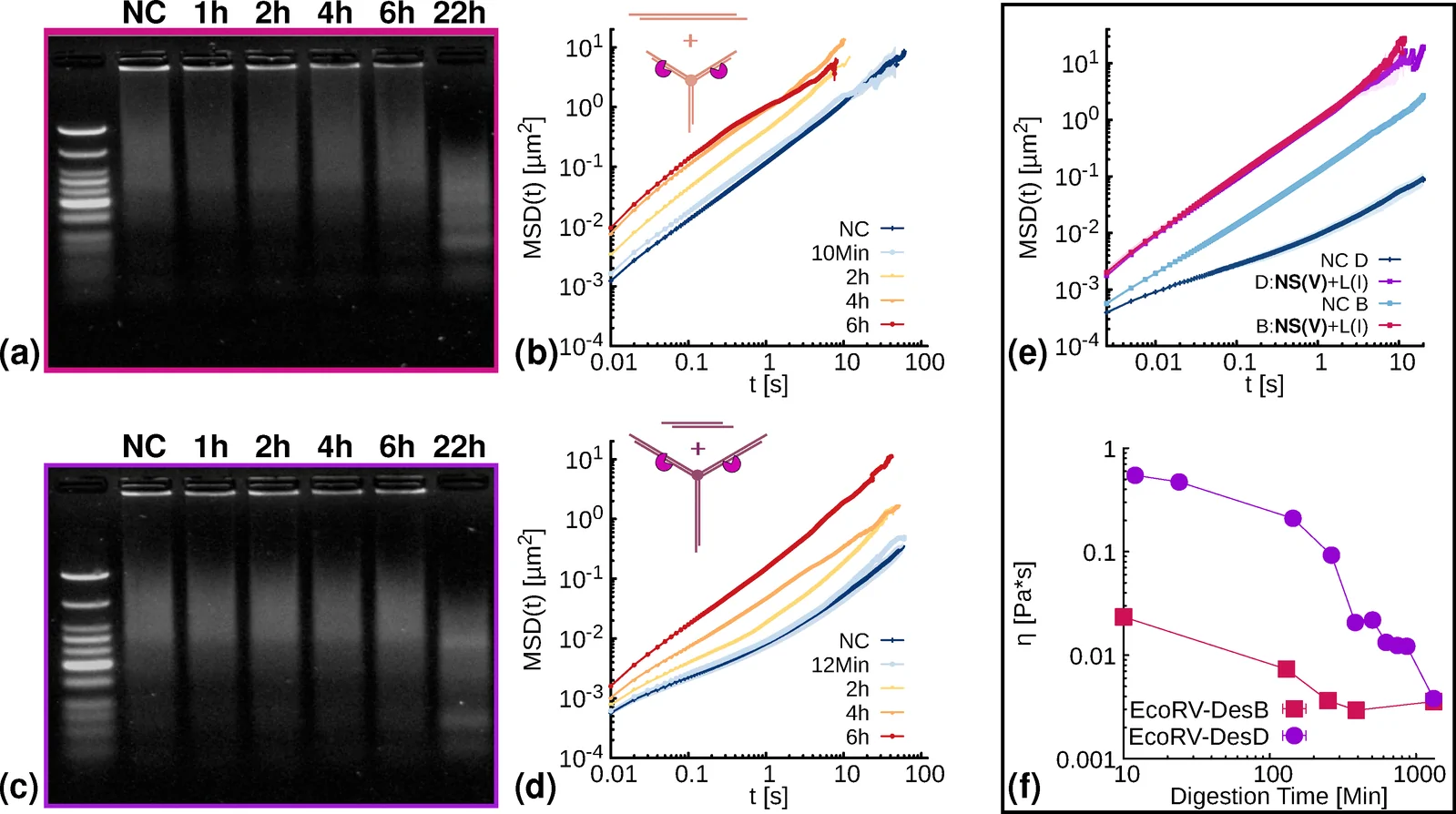
EcoRV digestion of DNAns designs B and D. (a) Agarose gel electrophoresis (native conditions) of design B hydrogels digested by EcoRV (100 U, at 0.9 U µg^−1^) at 37 °C. Reactions were stopped at different time points (1 h, 2 h, 4 h, 6 h, and 22 h). (b) MSD from MR experiments of 500 nm beads over the first 6 h of digestion at 37 °C. (c) and (d) show analogous results as (a) and (b) for design D hydrogels. (e) MSD curves comparing NC with samples digested for 22 h for both designs. (f) Viscosity as function of digestion time.

Gel electrophoresis at multiple time points (1, 2, 4, 6, and 22 hours) revealed limited digestion within the first 6 h for both designs, as DNA remained near the wells, but complete degradation occurred after 22 h (Fig. 5(a and c)). The degradation can be tracked more precisely with microrheology, which showed that even before full digestion, the MSD of tracer particles increased substantially within the first few hours, reflecting progressive network softening during partial cleavage (Fig. 5(b and d)).

Interestingly, the two designs displayed markedly different mechanical responses even before digestion. As shown in Fig. 5(e), design D (with longer arms) exhibited a near-plateau in the MSD at early times, reflecting a more elastic-like behavior, whereas design B appeared more liquid-like. Viscosity measurements confirmed this difference: design D initially had a viscosity about an order of magnitude higher than design B, but both converged to similar final values after 22 h digestion (Fig. 5(f)). The normalized viscosity ratios, *η*_22 h_/*η*_0_ = 0.006 for D and *η*_22 h_/*η*_0_ = 0.13 for B, show that D undergoes a 21-fold greater viscosity reduction compared to B. This difference is not only due to variations in enzymatic cleavage efficiency, but rather to the distinct initial architectures of the hydrogels. Together, these results reveal that the temporal evolution of viscoelasticity, and thus the effective degradation kinetics, can be modulated through DNAns geometry.

### 2.5. Sequence-specific hydrogel degradation enables controlled antibody release

**Fig. 6:**
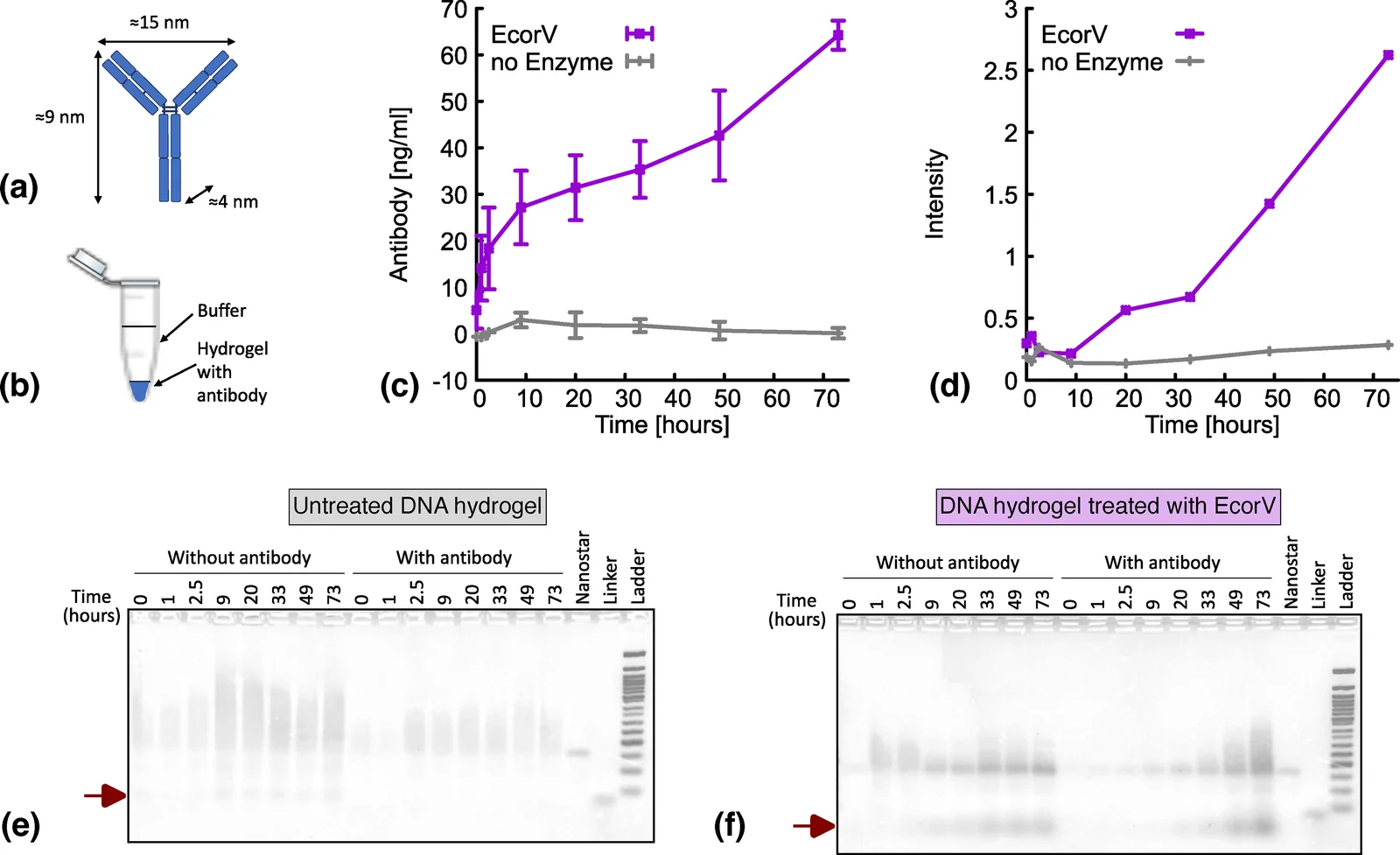
Antibody encapsulation, retention, and enzyme-mediated release in design D hydrogels. (a) Schematic of IgG antibody dimensions.^34^ (b) Antibodies are pre-mixed with DNAns hydrogel. Buffer is added on top of hydrogel sample to measure release over time. (c) Antibody release profiles measured by ELISA for hydrogels treated with EcoRV or no enzyme. (d) Quantification of DNA digestion products in the presence of antibody from the lowest-molecular-weight band in agarose gel electrophoresis (native conditions). (e) and (f) Representative gel images of hydrogels with and without antibody following treatment with no enzyme and EcoRV, respectively. The arrow indicates the position in the gel of the lowest molecular weight products.

To investigate the application of sequence-specific hydrogel degradation for controlled cargo release, we employed design D nanostars, which form hydrogels with pore size ≃34 nm that is large enough to accommodate Bevacizumab (approximately 15 nm × 9 nm × 4 nm in size,^34^ see Fig. 6(a)). Bevacizumab, an anti-VEGF monoclonal immunoglobulin G (IgG) currently in clinical trials as a treatment for cancer, was chosen as a model antibody. Since design D DNAns alone do not form a hydrogel in the absence of a linker, this allows us to pre-mix IgG with DNAns in the liquid state and subsequently induce gelation by adding the linker. This strategy facilitates antibody encapsulation without the need for heating, vigorous mixing, or vortexing, which could damage either the hydrogel or the cargo.

To assess antibody retention, DNA hydrogels pre-loaded with antibody were incubated without the addition of any restriction enzyme. DNAns buffer was then applied to the surface of each gel (schematically illustrated in Fig. 6(b)), and the supernatant was collected at specified time points over 48 hours. Antibody concentrations in the collected buffer were quantified by enzyme-linked immunosorbent Assay (ELISA), revealing negligible passive release, with antibody levels not exceeding 2 ng mL^−1^ within 48 hours (see Fig. S6). Detailed descriptions of sample preparation and experimental procedures are provided in the Methods section and SI Section SVIII.

We next investigated whether sequence-specific hydrogel degradation could trigger antibody release. Antibody-loaded hydrogels were incubated with EcoRV or left untreated as controls, each in triplicates. ELISA measurements of the collected supernatant (Fig. 6(c)) revealed negligible antibody release from untreated hydrogels over the duration of the experiment, whereas treatment with EcoRV resulted in a pronounced increase in antibody release, with concentrations reaching 64 ng mL^−1^ after 73 hours. These results demonstrate that restriction enzyme-mediated cleavage of the hydrogel network provides an effective trigger for the controlled release of encapsulated antibodies. We note, however, that the biological activity of the released antibody was not assessed in the present study. Although EcoRV specifically cleaves DNA and is not expected to directly affect antibody structure or function, and antibodies have previously been shown to retain their biological activity within DNA nanostar hydrogels,^35^ preservation of antibody activity after release remains to be experimentally verified.

To directly assess hydrogel degradation, supernatant samples collected at different time points, in the presence and absence of antibody, were analyzed by native agarose gel electrophoresis (Fig. 6(e and f)). The appearance and increasing intensity of the low-molecular-weight DNA band, corresponding to cleavage products released into the supernatant, confirm progressive EcoRV-mediated degradation of the hydrogel network (Fig. 6(d)). These observations qualitatively follow the same temporal trend as the antibody release profiles measured by ELISA (Fig. 6(c)), supporting the conclusion that sequence-specific degradation of the hydrogel network triggers antibody release. Establishing a quantitative relationship between the extent of hydrogel degradation and the fraction of antibody released remains an important direction for future work.

## 3. Conclusions

We have shown that molecular design governs both the susceptibility of DNA nanostar hydrogels to sequence-specific enzymatic degradation and their mechanical response. Under the conditions explored in this study, design A hydrogels are resistant to restriction endonuclease digestion (Fig. 2 and 3), whereas relocating the restriction sites farther from the nanostar core, onto linker strands, enables efficient hydrogel softening and degradation (Fig. 4). Moreover, DNAns geometry determines the initial viscoelastic properties of the hydrogels and modulates their mechanical response following enzymatic cleavage (Fig. 5). Finally, we show that DNAns hydrogels can retain antibodies and that sequence-specific degradation of an appropriate nanostar design can be harnessed to trigger the sustained release of encapsulated antibodies (Fig. 6).

Taken together, these results establish molecular design as the key determinant of both the material properties of DNAns hydrogels and their susceptibility to sequence-specific enzymatic degradation. The design principles identified here provide a framework for engineering DNA-based hydrogels with tailored mechanical properties, controlled enzymatic responsiveness, and cargo-release capabilities for *in vivo* applications, including antibody and drug delivery.

## 4. Methods

### 4.1. DNA nanostar design

We designed four DNA nanostar variants (A–D) with distinct structural features (see Fig. 1). Each nanostar is assembled by annealing three ssDNA oligonucleotides (49 nt for A, 50 nt for B, 48 nt for C, and 74 nt for D; sequences in SI, Tables S1–S4). Equimolar amount of oligos were mixed in nanostar buffer ([NaCl] = 150 mM, [Tris] = 40 mM, [Acetate] = 40 mM, [EDTA] = 1 mM, pH 8.0) and cooled from 90 °C to room temperature over 4 h to yield three-armed nanostars.^4^

Design A forms self-complementary 6-nt sticky ends (5'-CGATCG-3'), allowing any arm to hybridize with a single arm of another nanostar. Unpaired adenines at the core and before the sticky ends provide flexibility at both the central junction and inter-nanostar connection.^18,24^

Designs B–D use linkers to mediate assembly. Each nanostar arm terminates in an 8-nt non-palindromic sticky end (5′-CGATTGAC-3′) that hybridizes to a complementary linker.^17^ Linkers are formed by annealing two ssDNAs in 150 mM NaCl. Mixing DNAns and linkers at a 1 : 1.5 molar ratio produces hydrogels. Unpaired adenines are retained at the core in designs B and D but omitted in C, resulting in a more rigid structure. In designs B-D there are no unpaired adenines before the the sticky ends, resulting in a more rigid bond between the DNAns and the linker.

Melting temperatures of the nanostars (*T*_m1_), linkers (*T*_m2_), and sticky ends (*T*_*m*3_) were computed using NUPACK^22^ under digestion conditions ([NaCl] = 150 mM, [Mg^2+^] = 10 mM, [ssDNA] = 250 µM), ensuring *T*_m1_ > *T*_m3_ and *T*_m2_ > *T*_m3_ by design (Fig. 1e).

### 4.2. Restriction enzymes

To study hydrogel degradation, nanostar arms were engineered to include sequences recognized by REs, illustrated as “pac-man” symbols in Fig. 1 (EcoRI-HF in green, BtgI in red, MspA1I in cyan, and EcoRV-HF in magenta; “HF” denotes high-fidelity, omitted for brevity in the main text).

In design A, two arms carry EcoRI sites, while the third contains one BtgI and one MspA1I site (Fig. 1a). Designs B–D use EcoRI and EcoRV in complementary configurations: if one enzyme site is on the nanostar, the other is placed on the linker (Fig. 1b–d). Full oligo sequences are provided in the SI.

The enzymes used fall into three categories: (i) sticky-end cutters (EcoRI-HF, BtgI) that generate 4-nt overhangs; (ii) blunt cutters (MspA1I, EcoRV-HF) that leave no overhangs; and (iii) the non-specific endonuclease DNaseI. All enzymes were obtained from New England Biolabs (NEB), which defines 1 unit (U) as the amount of enzyme required to digest 1 µg of substrate DNA at 37 °C in a 50 µL reaction within 1 h (10 min for DNaseI). For comparison, in our microrheology experiments, we use !100 µg of DNA in a 10 µL reaction at 37 °C.

In all designs, the melting temperature of sticky-end hybridization exceeds the enzymes’ optimal working temperature ((37°), ensuring network integrity under digestion conditions. Reported enzyme half-lives at (37° are 2–4 h (BtgI, EcoRV-HF), 4–8 h (MspA1I), and >8 h (EcoRI-HF).^36^

### 4.3. Gel electrophoresis

Gel electrophoresis was performed on 3% agarose gels pre-stained with SYBR Safe (1 : 10 000 dilution from stock). For design A, gels were prepared by dissolving 3 g agarose (Sigma-Aldrich) in 100 mL of 1× LAB buffer (lithium acetate borate). For designs B–D, 1.5 g agarose was dissolved in 50 mL of 1× TAE buffer (Tris, Acetate, EDTA). Positive and negative controls, as well as reaction samples, were prepared following standard protocols (see SI, Sections SIII and SIV).

Gel electrophoresis was carried out at room temperature using 150 V (∼7.5 V cm^−1^ using a tray with 20 cm between electrodes) for 35 min for design A and 50 V (∼3.3 V cm^−1^) for 70 min for designs B–D. DNA bands were visualized under UV illumination (Syngene GBox) and compared against a low molecular weight DNA ladder (NEB).

### 4.4. Particle tracking microrheology (PTMR)

Unless stated otherwise, microrheology experiments were performed at a DNAns concentration of 250 µM (12.2 mg mL^−1^ DNA) in 10 µL total volume. Sample preparation followed the procedure described in the Gel Electrophoresis: sample preparation section of the SI.

Polystyrene tracer beads (200 nm or 500 nm diameter, Sigma-Aldrich) were mixed in the sample. To minimize drift and evaporation, ∼100 µm sticky spacers were used to seal the sample between a glass slide and coverslip. Solutions were equilibrated for 5 min at room temperature in a stage-top temperature-controlled chamber (OKO Lab) mounted on an inverted Nikon microscope equipped with a 100× oil-immersion objective (bright-field mode). Two acquisition modes were employed: (i) low-frame-rate tracking of 20–30 beads within a 1024 × 1024 px field of view at 100 fps for 2 min, repeated every 10 min to monitor temporal evolution; and (ii) high-frame-rate tracking of 5–10 beads within a 512 × 512 px field at 400 fps for 10 s, also repeated every 10 min.

Particle trajectories were extracted using trackpy (https://github.com/soft-matter/trackpy) and analyzed with custom C++ scripts to compute mean-squared displacements. The diffusion coefficient, *D*_p_, was obtained from the linear regime of the MSD (MSD = 2*dD*_p_*t*), where *d* is the dimensionality. The sample viscosity, *η*, was then calculated using the Stokes–Einstein relation, *η* = *k*_B_*T*/(3π*D*_p_*a*) where *k*_B_ is the Boltzmann constant, *T* the absolute temperature, *a* the tracer radius, and *D*_p_ the diffusion coefficient derived from MSD analysis.

### 4.5. ELISA

Antibody release was analysed with an enzyme-linked immunosorbent assay (ELISA) for quantitative detection of human IgG using a Human IgG (Total) Uncoated ELISA Kit (Invitrogen, 88-50550) in accordance with the manufacturer's instructions. Bevacizumab (BioRad, MCA6089) was used as the human IgG standard. Absorbance at 450 nm was measured with a Tecan Infinite® M200 with i-control^TM^ software, v2.0, hosted by the Edinburgh Genome Foundry.

For antibody release experiments, design D hydrogels were prepared with a final antibody concentration of 0.034 µg µL^−1^. Detailed hydrogel preparation, washing, and ELISA protocols are provided in the SI (Section SVIII).

## Conflicts of interest

There are no conflicts to declare.

## Supplementary Material

SM-022-D6SM00563B-s001

SM-022-D6SM00563B-s002

## Data Availability

The gel electrophoresis and microrheology data supporting this article, including mean squared displacement datasets, are available at https://git.ecdf.ed.ac.uk/ygutier2/data-dnans-deg. Supplementary information (SI): Document with details of the experiments described in the main text are provided. See DOI: https://doi.org/10.1039/d6sm00563b.
