# Supplementary material for: Designing DNA nanostar hydrogels for sequence-specific degradation and antibody release
Source: Soft Matter. 2026 Aug 5;22(32):5401–10. doi: 10.1039/d6sm00563b (PMC13439265; doi:10.1039/d6sm00563b)
Supplement: Supplementary file 1 [file SM-022-D6SM00563B-s001.pdf]

Supplementary Information (SI) for Soft Matter.
This journal is © The Royal Society of Chemistry 2026

Soft Matter

ARTICLE TYPE

Cite this: DOI: 00.0000/xxxxxxxxxx

Supplementary Information: Designing DNA nanostar hydrogels for sequence-specific degradation and antibody release

Giorgia Palombo,[a] Christine A. Merrick,[b] Jennifer Harnett,[a] Susan Rosser,[b] Davide Michieletto,[a,c,d,*] and Yair Augusto Gutiérrez Fosado[a*]

Contents

I Design specifications of DNA nanostars 2

II DNA Nanostars and Linkers assembly protocol 4

III Sample Preparation for gel electrophoresis and microrheology 4
- Positive Control . . . 5
- Reaction Solution . . . 5

IV Gel Electrophoresis 5

V Confocal Microscopy 6
- Specimen preparation . . . 6
- RE-dependent DNA Nanostar droplet degradation . . . 7

VI Effect of glycerol on the rheology of DNA nanostars 7

VII Non-specific enzyme DNase I digests all DNAns gel designs 8

VIII ELISA 8

[a] *School of Physics and Astronomy, University of Edinburgh, Peter Guthrie Tait Road, Edinburgh, EH9 3FD, UK.*
[b] *Centre for Engineering Biology, School of Biological Sciences, University of Edinburgh, Edinburgh, UK.*
[c] *MRC Human Genetics Unit, Institute of Genetics and Cancer, University of Edinburgh, UK.*
[d] *International Institute for Sustainability with Knotted Chiral Meta Matter (WPI-SKCM^2), Hiroshima University, Higashi-Hiroshima, Hiroshima 739-8526, Japan*

I Design specifications of DNA nanostars

As mentioned in the main text, we designed four distinct types of DNAns building blocks using NUPACK[1]. Each DNAns was assembled from three single-stranded DNA (ssDNA) oligonucleotides (oligos). Upon hybridization, these oligos form a three-armed (Y-shaped) structure (see Fig. **1**).

In design A, each arm consists of a 20 base-pair (bp) segment terminating in a self-complementary 6-nucleotide (nt) fragment (5'-CGATCG-3'). This sticky end is identical on all three arms, allowing any arm of one nanostar to hybridize with any (but only one) arm of another nanostar. Unpaired adenines are introduced as flexible joints (FJ) at two positions: at the core of the Y-shaped structure (FJ1) and before the sticky ends (FJ2). These flexible regions enhance the overall flexibility of both the nanostar and its interconnections[2,3]. The specific sequences used are provided in Table S1, which also include the recognition sites for the restriction enzymes (REs).

For designs B–D, each DNAns arm (20 bp long for B and C, and 32 bp long for D) terminates in a non-self-complementary 8 nt sticky end sequence, 5'-CGATTGAC-3'. This sequence was specifically chosen to prevent self-assembly between DNAns[4]. Instead, these sticky ends are complementary to those on the linker (L), which contains two overhangs that mediate hybridization between different nanostars. The linker is a linear double-stranded DNA segment whose length varies among designs: 32 bp for designs B and C, and 20 bp for design D.

Additionally, in designs B-D, there are no unpaired adenines before the sticky ends, resulting in a more rigid connection between the DNAns and the linker. However, the unpaired adenines at the core are retained in designs B and D, but omitted in design C, leading to an overall more rigid structure. The sequences corresponding to these designs are detailed in Tables S2, S3, and S4.

	Design A: NS(**EcoRI**, **MspAI**, **BtgI**)
NS1	5′−GTCAATGC*CCGCGG*GCAATT/**AA**/GGGCATG**GAATTC**CGCATCC/**A**/CGATCG−3′
NS2	5′−GGATGCG**GAATTC**CATGCCC/**AA**/CCCTTGG**GAATTC**GATCCCG/**A**/CGATCG−3′
NS3	5′−CGGGATC**GAATTC**CCAAGGG/**AA**/AATTGC*CCGCGG*GCATTGAC/**A**/CGATCG−3′

Table S1 Design A sequences: Each oligo is 49 nt long, with a FJ1 at the core (/**AA**/) and an additional FJ2 motif preceding the sticky ends (/**A**/). The sticky end is 6 nt long (CGATCG). Restriction enzyme sites are indicated as: EcoRI (green), BtgI (red) and MspA1I (blue box).

	Design B: NS(**EcoRI**) + L(**EcoRV**)
NS1	5′−GTCAATGCCCGCGGGCAATT/**AA**/GGGCATG**GAATTC**CGCATCC/CGATTGAC−3′
NS2	5′−GGATGCG**GAATTC**CATGCCC/**AA**/CCCTTGG**GAATTC**GATCCCG/CGATTGAC−3′
NS3	5′−CGGGATC**GAATTC**CCAAGGG/**AA**/AATTGCCCGCGGGCATTGAC/CGATTGAC−3′
L1	5′−CCTATTCGCATGA**GATATC**CATTCACCGTAAG/GTCAATCG−3′
L2	5′−CTTACGGTGAATG**GATATC**TCATGCGAATAGG/GTCAATCG−3′
	Design B: NS(**EcoRV**) + L(**EcoRI**)
NS1	5′−GTCAATGCCCGCGGGCAATT/**AA**/GGGCATG**GATATC**CGCATCC/CGATTGAC−3′
NS2	5′−GGATGCG**GATATC**CATGCCC/**AA**/CCCTTGG**GATATC**GATCCCG/CGATTGAC−3′
NS3	5′−CGGGATC**GATATC**CCAAGGG/**AA**/AATTGCCCGCGGGCATTGAC/CGATTGAC−3′
L1	5′−CCTATTCGCATGA**GAATTC**CATTCACCGTAAG/GTCAATCG−3′
L2	5′−CTTACGGTGAATG**GAATTC**TCATGCGAATAGG/GTCAATCG−3′

Table S2 Design B sequences: Each NS oligo is 50 nt long, with a FJ1 at the core (/**AA**/). The NS sticky end is 8 nt long (CGATTGAC). Linker oligos are 40 nt long, each with an 8 nt sticky end (GTCAATCG), which is complementary to that of the NS. RE sites are labelled as follows: EcoRI (green) and EcoRV (magenta). The oligo sequences with the RE sites inverted between NS and L are also reported.

	Design C: NS(**EcoRI**)+ L(**EcoRV**)
NS1	5′–GTCAATGCCCGCGGGCAATTGGGCATG**GAATTC**CGCATCC/CGATTGAC–3′
NS2	5′–GGATGCG**GAATTC**CATGCCCCCTTGG**GAATTC**GATCCCG/CGATTGAC–3′
NS3	5′–CGGGATC**GAATTC**CCAAGGGAATTGCCCGCGGGCATTGAC/CGATTGAC–3′
L1	5′–CCTATTCGCATGA**GATATC**CATTCACCGTAAG/GTCAATCG–3′
L2	5′–CTTACGGTGAATG**GATATC**TCATGCGAATAGG/GTCAATCG–3′
	Design C: NS(**EcoRV**) + L(**EcoRI**)
NS1	5′–GTCAATGCCCGCGGGCAATTGGGCATG**GATATC**CGCATCC/CGATTGAC–3′
NS2	5′–GGATGCG**GATATC**CATGCCCCCTTGG**GATATC**GATCCCG/CGATTGAC–3′
NS3	5′–CGGGATC**GATATC**CCAAGGGAATTGCCCGCGGGCATTGAC/CGATTGAC–3′
L1	5′–CCTATTCGCATGA**GAATTC**CATTCACCGTAAG/GTCAATCG–3′
L2	5′–CTTACGGTGAATG**GAATTC**TCATGCGAATAGG/GTCAATCG–3′

Table S3 Design C sequences. Each NS oligo is 48 nt long and lacks the FJ1 at the core. The NS sticky end is 8 nt long(CGATTGAC). L oligos (40 nt) contain the complementary sticky end (GTCAATCG) to the NS. Restriction enzyme sites are indicated: EcoRI (green) and EcoRV (magenta). Sequences with inverted RE sites between NS and L are also reported.

	Design D: NS(**EcoRI**) + L(**EcoRV**)
NS1	5′–CGTAAGGTCAATGCCCGCGGGCAATTCCTATT/**AA**/CGTAA GGGGCATG**GAATTC**CGCATCCTATTCC/CGATTGAC–3′
NS2	5′–GGAATAGGATGCG**GAATTC**CATGCCCCTTACG/**AA**/CCTAT TCCCTTGG**GAATTC**GATCCCGCGTAAG/CGATTGAC–3′
NS3	5′–CTTACGCGGGATC**GAATTC**CCAAGGGAATAGG/**AA**/AATAG GAATTGCCCGCGGGCATTGACCTTACG/CGATTGAC–3′
L1	5′–CGCATGA**GATATC**CATTCAG/GTCAATCG–3′
L2	5′–CTGAATG**GATATC**TCATGCG/GTCAATCG–3′
	Design D: NS(**EcoRV**) + L (**EcoRI**)
NS1	5′–CGTAAGGTCAATGCCCGCGGGCAATTCCTATT/**AA**/CGTAA GGGGCATG**GATATC**CGCATCCTATTCC/CGATTGAC–3′
NS2	5′–GGAATAGGATGCG**GATATC**CATGCCCCTTACG/**AA**/CCTAT TCCCTTGG**GATATC**GATCCCGCGTAAG/CGATTGAC–3′
NS3	5′–CTTACGCGGGATC**GATATC**CCAAGGGAATAGG/**AA**/AATAG GAATTGCCCGCGGGCATTGACCTTACG/CGATTGAC–3′
L1	5′–CGCATGA**GAATTC**CATTCAG/GTCAATCG–3′
L2	5′–CTGAATG**GAATTC**TCATGCG/GTCAATCG–3′

Table S4 Design D sequences: Each NS oligo is 74 nt long and includes a FJ1 (/**AA**/). The NS sticky end is 8 nt long (CGATTGAC). L oligos (28 nt long) contain a sticky end (GTCAATCG), which is complementary to that of the NS. RE sites are indicated: EcoRI (green) and EcoRV (magenta). The oligo sequences with the RE sites inverted between NS and L are also reported.

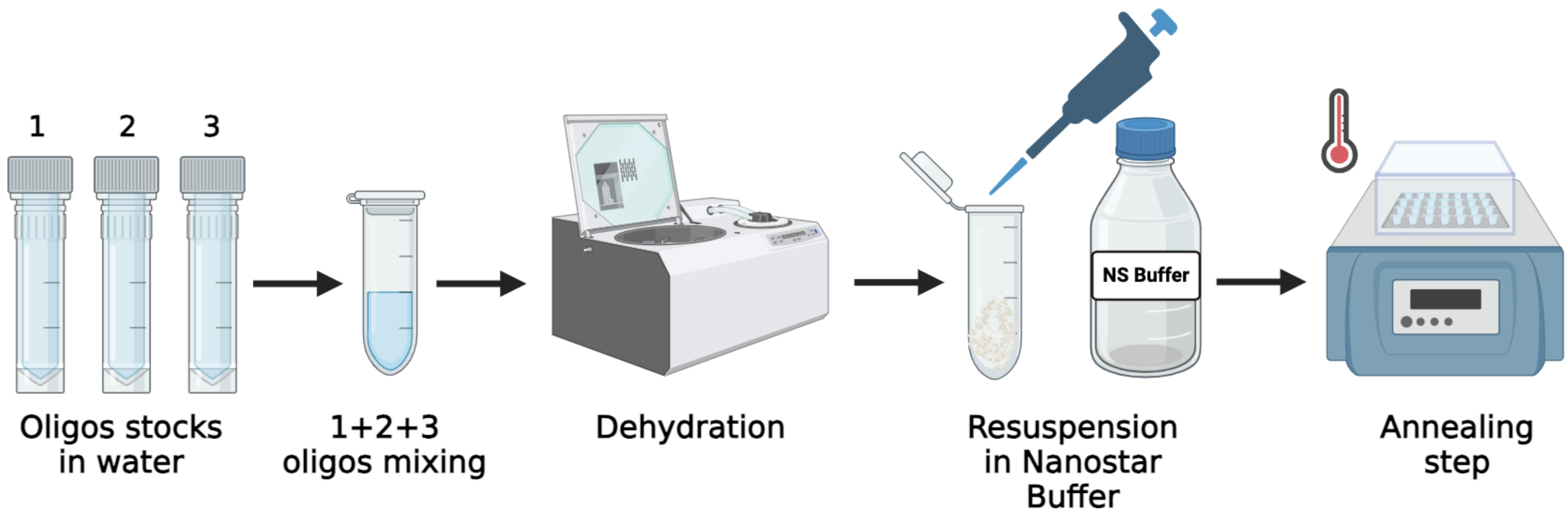

Fig. S1 DNA Nanostar assembly. Workflow for preparing nanostar samples (created with BioRender).

II DNA Nanostars and Linkers assembly protocol

DNA oligonucleotides were purchased in dehydrated form from Integrated DNA Technologies (IDT). Following the provider's recommendations, tubes were centrifuged for 30 s to collect all material at the bottom. The oligos were rehydrated with Ultrapure Water to prepare 1 mM stock solutions (see Fig. S1), which were typically stored in a refrigerator.

Equimolar amounts of the three oligos were combined from the stock solutions into a new Eppendorf® DNA LoBind® tube. The mixture was dried at 60°C for approximately 2 h in a vacuum concentrator (Eppendorf, Concentrator plus), with the tube open but covered by a sterile filter to prevent contamination. Once fully dehydrated, the sample was resuspended in Nanostar buffer (40 mM Tris, 40 mM sodium acetate, 1 mM EDTA, pH 8.0, and 150 mM NaCl)[5]. The buffer volume added was adjusted to reach the desired final concentration.

To ensure complete dissolution of the sample, alternating cycles of heating at 60°C and vortexing were applied until no dry DNA was visible. The annealing process was then performed by heating the solution to 90°C ($T > T_{m1}$) for 2 min in a well-insulated heat block, followed by gradual cooling to 25°C ($T < T_{m3}$) over approximately 4 h. This slow cooling step was crucial for the correct assembly of the Y-shaped DNA structure. The resulting DNA nanostar solution was stored at 4°C until use.

Before experiments, the sample concentration was verified by measuring the absorbance at 260 nm (A_{260}) using a UV spectrophotometer (Thermo Scientific, NanoDrop Lite). Dilutions of 1:100 ($A_{260,1\%}$) and 1:1000 ($A_{260,0.1\%}$) were prepared in water, assuming that the absorbance of the 0.1% dilution corresponded solely to ssDNA. Concentrations were calculated as described in references[5,6].

For linker formation in designs B-D, we prepared the stock solution in a separate tube from the DNAns. Equimolar amounts of the two ssDNA oligos were mixed in water, dehydrated, and resuspended in Nanostar buffer at a final concentration of 2 mM. The linker solution was annealed by cooling from 90 to 25°C to yield linear dsDNA. Its concentration was determined using a NanoDrop after 1:10 and 1:100 dilutions in Nanostar buffer. Mixing DNAns B-D with their corresponding linker at a 1:1.5 molar ratio[4] produced a hydrogel through hybridization of complementary sticky ends.

III Sample Preparation for gel electrophoresis and microrheology

Standardized protocols were developed to ensure experimental consistency when investigating the behavior of the four DNAns designs, with and without restriction enzymes (REs), under various conditions. Three types of samples were prepared: (i) a Positive Control (PC) with low DNA concentration to verify the experimental setup; (ii) a Negative Control (NC) at both low and high DNA concentrations to confirm that observed effects originated from RE activity rather than external factors; and (iii) a Reaction Solution (RS) with high DNA concentration to evaluate RE efficiency in more concentrated or gel-like conditions.

Unless otherwise stated, enzymes were used at the highest stock concentrations available from the supplier (NEB): 2 U/μL for DNase I, 10 U/μL for MspA1I and BtgI, and 100 U/μL for EcoRI-HF and EcoRV-HF.

We note that enzyme activity units are defined under assay-specific conditions. In particular, 1U of DNaseI corresponds to complete degradation of 1 μg of pBR322 DNA within 10 min at 37°C, whereas 1U of BtgI corresponds to digest 1 μg of pBR322 DNA in 1 hour at 37°C in a total reaction volume of 50 μl. For the rest of the site-specific REs used here, 1U is defined as the amount required to digest 1 μg of $\lambda-$DNA in 1h under standard conditions. Because these definitions rely on different substrates and cleavage modes (non-specific versus sequence-specific), the nominal activity does not directly reflect cleavage efficiency on other DNA sequences. Accordingly, comparisons are based on observed activity under the experimental conditions employed.

Positive Control

Positive control samples were prepared following the guidelines in the "Optimizing Restriction Endonuclease Reactions" section of the NEB website[7]. This protocol was used for gel electrophoresis experiments (see Fig. S3).

Each 50 μL reaction contained a final DNA concentration of 0.4 μM (in the phase-separation region), prepared by mixing in a single Eppendorf tube: (i) 38 μL of ultrapure water, (ii) 1 μL of DNA (DNAns or linker, at 1 μg/μL), (iii) 5 μL of 10× Nanostar Buffer to maintain constant ionic strength, (iv) 5 μL of 10× enzyme reaction buffer (rCutsmart for REs or DNaseI Reaction Buffer for DNaseI), and (v) 1 μL of the enzyme (replaced by the corresponding enzyme diluent buffer for the NC). The mixture was vortexed briefly and incubated in a heat block at 37 °C for 1 h.

To stop digestion, 10 μL of 6× Purple Gel Loading Dye containing 60 mM EDTA was added to both PC and NC samples. The prepared solutions were then loaded into agarose gel wells for electrophoresis.

Reaction Solution

A different protocol was established for reaction samples prepared at higher DNAns concentrations, which was also applied to microrheology experiments.

For DNAns Design A, samples were prepared at 250 μM DNAns (12.1 μg/μL) and incubated with different enzymes and units: 2 U of DNaseI, 20 U or 100 U of EcoRI-HF, 10 U of MspA1I, and 10 U of BtgI, in a final volume of 10 μL. These corresponded to enzyme-to-DNA mass ratios of 0.02 U/μg (DNaseI), 0.2 U/μg or 0.8 U/μg (EcoRI), and 0.08 U/μg (MspA1I and BtgI). The highest enzyme units available from NEB were used. Each reaction was prepared by incubating an Eppendorf tube in a 60 °C heat block and sequentially adding: (i) 5 μL of DNAns at 500 μM (24.4 μg/μL) pre-heated at 60°C for 2 minutes, (ii) 2.5 μL of 1× Nanostar Buffer, (iii) 1 μL of enzyme reaction buffer, (iv) 1 μL of enzyme (or diluent for NC), and (v) 0.5 μL of water (replaced by polystyrene beads in microrheology experiments). Due to the viscosity of the solutions, mixing was performed by pipetting 3–5 times using tips preheated to 60 °C to prevent premature cooling. The samples were incubated at 37 °C for 8–16 h, depending on enzyme stability. To stop digestion, 2 μL of 6× Purple Gel Loading Dye was added to each tube. Before opening, samples were briefly centrifuged to collect condensate at the bottom. Both NC and RS samples were then diluted in Nanostar Buffer to 25 ng/μL, and 10 μL of each (corresponding to 250 ng total DNA) was loaded per gel well.

For Designs B-D, reactions were conducted at different enzyme-to-DNA ratios (0.45, 0.7, and 0.9 U/μg) by varying the total DNAns and linker mass while keeping enzyme units fixed at 100 U for EcoRI and EcoRV (maximum available from NEB). This allowed determination of the minimum enzyme-to-DNA ratio required for digestion at high DNA concentrations. Unlike Design A, these systems included linkers that enabled lower DNAns concentrations while preserving viscoelastic properties[4]. Two stock solutions were first prepared: 1 mM Nanostar (NS) and 2 mM Linker (L) in Nanostar Buffer. Both remained liquid at room temperature because their sticky ends were non-complementary. Gels (NS+L) were then formed by mixing NS and L in specific ratios (1:1.5[4]) to reach desired final concentrations within a total volume of 10 μL. For example, to achieve 0.45 U/μg, 3.3 μL of NS, 2.5 μL of L, and 4.2 μL of Nanostar Buffer were combined, yielding 0.33 mM NS and 0.50 mM L. For 0.68 U/μg, final concentrations were 0.23 mM (NS) and 0.35 mM (L), and for 0.9 U/μg, 0.16 mM (NS) and 0.24 mM (L). The NS+L gels, which were highly viscous, were heated at 60 °C (including tips) for 2 min. Subsequently, 7.5 μL of the melted gel was mixed with (i) 1 μL enzyme reaction buffer, (ii) 1 μL enzyme (or diluent for NC), and (iii) 0.5 μL water (replaced by polystyrene beads in microrheology experiments). The samples were incubated at 37 °C for 21 h to optimize enzymatic digestion. To stop the reaction, 2 μL of 6× Purple Gel Loading Dye was added. The resulting samples were diluted in Nanostar Buffer to 25 ng/μL (if we consider only the mass of NS in the gel) or 45 ng/μL (considering the mass of both NS and L). Finally, 10 μL of each sample (250 or 450 ng total DNA) was loaded per gel well.

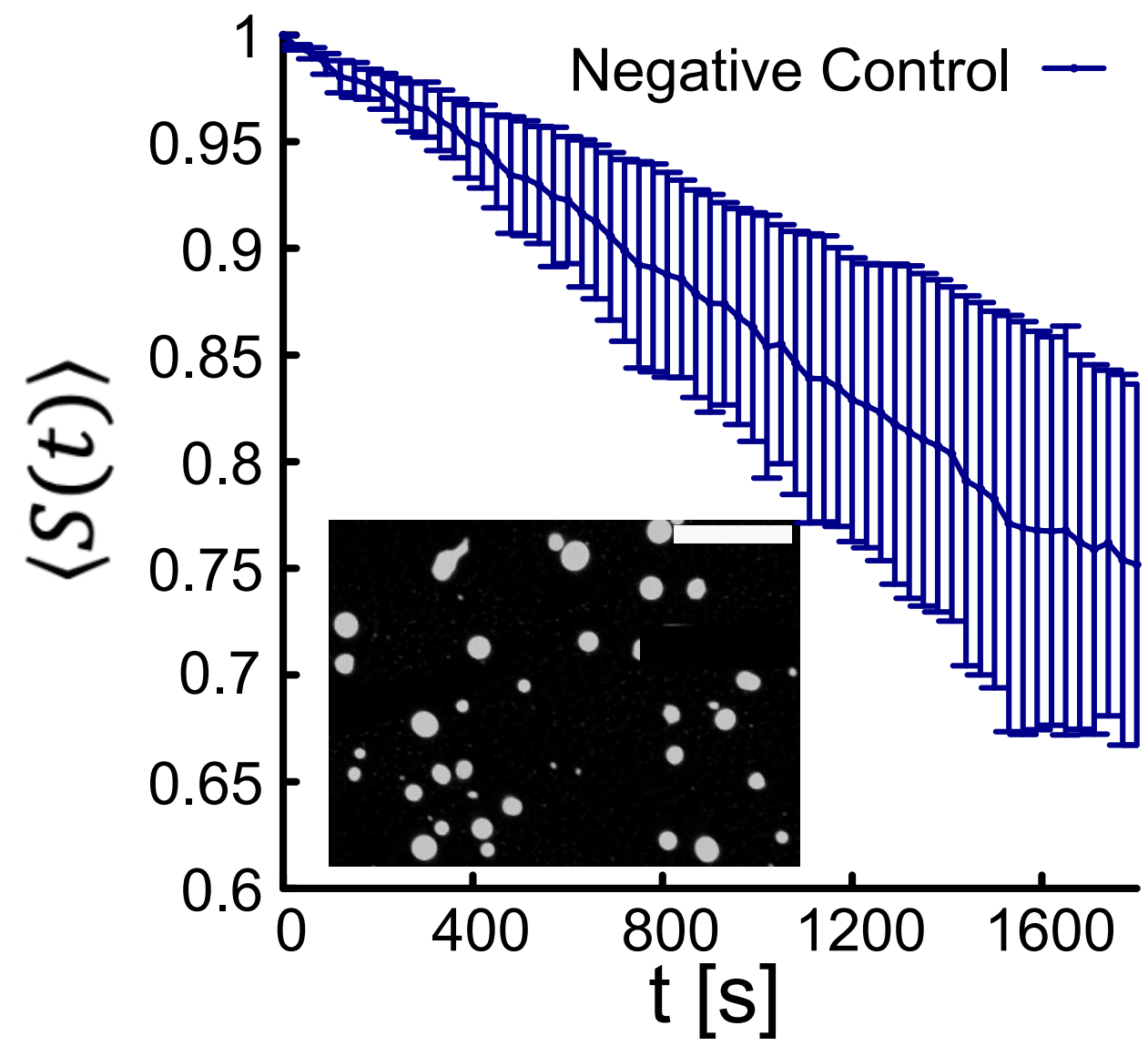

Fig. S2 Confocal imaging of DNAns droplets (Design A). YOYO-I–labelled droplets in a 2 μM solution before restriction enzyme addition (negative control; scale bar: 25 μm). The mean droplet size, $\langle S(t) \rangle$, decreases over time (t), indicating droplet shrinkage due to photobleaching.

IV Gel Electrophoresis

Gel electrophoresis is a widely used method for separating and analyzing charged DNA samples[8]. The technique relies on measuring the migration of DNA molecules under an electric field, which correlates with their size and topology[9,10]. Migration occurs through the pores of an agarose or polyacrylamide gel, whose pore size can be tuned by adjusting the polymer concentration. Smaller DNA fragments migrate faster and travel further than larger ones, as they experience less frictional resistance[8].

In this work, gel electrophoresis was performed using 3%

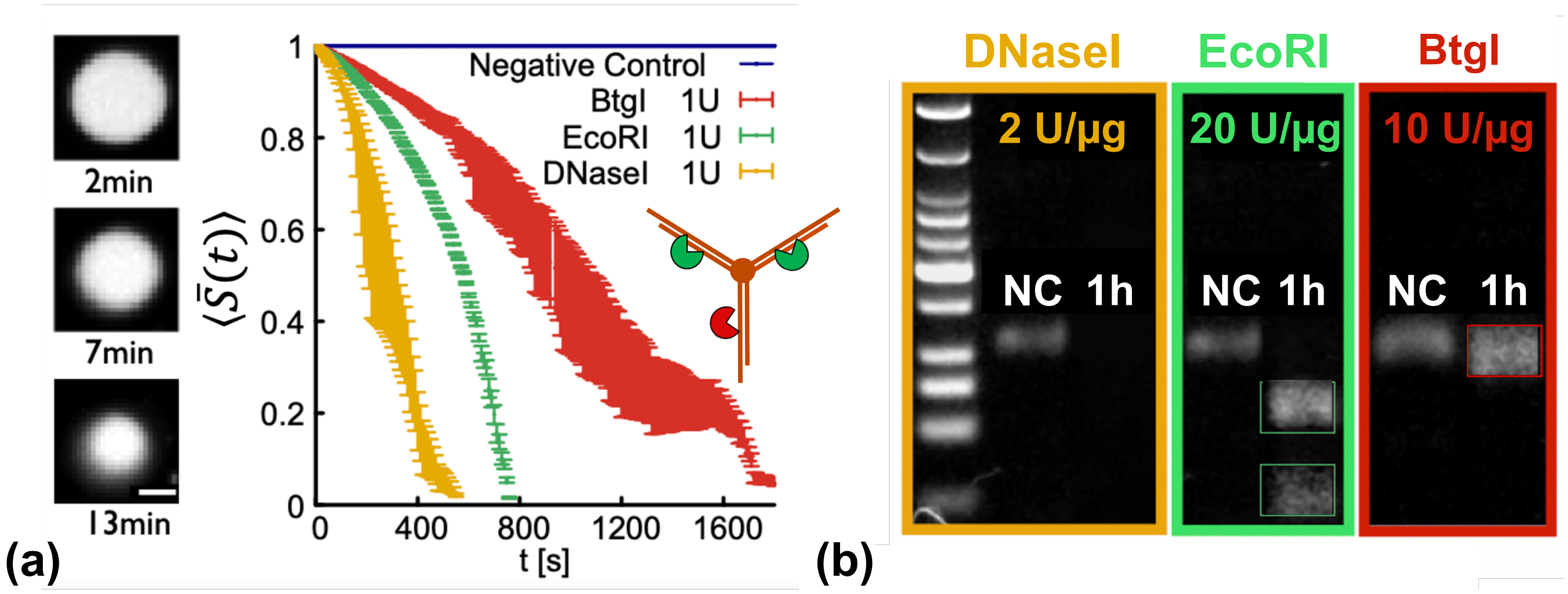

Fig. S3 RE-dependent DNAns cleavage within the phase boundary. (a) Left: Time-lapse confocal images showing the degradation of a YOYO-I–labeled droplet during EcoRI digestion (scale bar: 25 μm). Right: Normalized mean droplet size, $\langle \overline{S}(t) \rangle$, as a function of time. Degradation rates vary among enzymes: DNaseI (yellow) induces the fastest degradation, followed by EcoRI (green, two restriction sites) and BtgI (red, one restriction site), all resulting in complete digestion. Inset: Schematic of the three-arm DNAns A structure, showing a flexible core, flexible joints preceding 6-nt sticky ends, and 20-bp arms. **(b)** Gel electrophoresis of DNAns solutions incubated for 1 h with DNaseI (2 U/μg, yellow), EcoRI (20 U/μg, green), and BtgI (10 U/μg, red).

agarose gels to separate the small DNAns fragments. The gel matrix was prepared by dissolving UltraPure agarose powder in either TAE (Tris, acetic acid, EDTA) or LAB (lithium acetate borate) buffer, followed by heating until complete dissolution. The agarose solution was pre-stained with SYBR Safe dye (1:10,000 dilution) to enable visualization of DNA bands after electrophoresis. The molten solution was poured into a casting tray fitted with a comb to form wells and allowed to cool to room temperature.

Electrophoresis was carried out in a horizontal configuration at room temperature. After solidification, the gel was submerged in the same buffer used for its preparation (TAE or LAB) to ensure electrical conductivity and maintain stable pH. A voltage was applied across the gel, with the cathode at one end and the anode at the other. As DNA molecules are negatively charged they migrate toward the positive electrode. After electrophoresis, the separated DNA bands were visualized under a UV transilluminator and compared against a molecular weight marker. A low molecular weight DNA ladder (25–766 bp) was used, matching the expected sizes of the nanostars and linkers.

For experiments involving only DNAns (Design A), the agarose gel was prepared by dissolving 3 g of agarose in 100 mL of 1× LAB buffer. Electrophoresis was performed at 150 V for 35 min, corresponding to an electric field strength of 7.5 V/cm (calculated as 150V/20cm, where 20 cm is the electrodes separation). The use of high voltage is enabled by lithium ions in the LAB buffer, which possess large hydration shells and low electrokinetic mobility, thereby reducing electrical conductance and minimizing heat generation during electrophoresis[11].

For the other designs (B-D), a smaller gel tray was used, requiring 1.5 g of agarose in 50 mL of 1× TAE buffer. In this case, electrophoresis was conducted at 50 V for 70 min, corresponding to an electric field strength of 3.3 V/cm.

V Confocal Microscopy

The spatial distribution and structural characteristics of the system at low DNAns concentrations were examined using confocal microscopy. This advanced fluorescence imaging technique provides high-resolution images and enables optical sectioning of samples. Unlike conventional widefield fluorescence microscopes that illuminate the entire specimen simultaneously, confocal systems focus a laser beam on a specific point within the sample and collect only the emitted light by excited fluorophores from that focal plane. A pinhole placed before the detector blocks out-of-focus light, significantly enhancing optical resolution (up to ∼200 nm laterally and ∼600 nm axially[12]) and contrast. The size of the illuminated spot depends on the excitation wavelength, numerical aperture (NA) of the objective, and pinhole diameter.

In confocal microscopy, a laser scans a single optical plane by rastering the beam across the sample. By acquiring images at multiple focal depths (z-stacks), three-dimensional reconstructions of the sample can be generated. This capability is particularly valuable for studying thick or heterogeneous structures. Confocal systems can also image multiple fluorescent labels using distinct lasers and filters, allowing simultaneous visualization of different components within the same specimen.

All imaging was performed using a Zeiss LSM 700 Laser Scanning Microscope equipped with either a 40× oil-immersion objective (NA 1.3) or a 20× air objective (NA 0.4), depending on experimental requirements.

Specimen preparation

Our objective was to investigate the behavior of DNAns droplets formed at very low DNA concentrations, within the phase separation region[13,14]. We focused on Design A nanostars to evalu-

ate how the presence or absence of restriction enzymes influences droplet size, providing insight into the enzyme-responsive properties of the system. A stock solution was prepared by mixing 10 μL of DNAns (at 0.02 mM) with 1 μL of YOYO-I dye (1 mM) in a total volume of 100 μL. The mixture was gently rotated for 1 h to allow droplet formation while preventing sedimentation. Subsequently, 10 μL of the sample (with or without REs) was prepared in a separate Eppendorf tube and then transferred to a sealed coverslip chamber and allowed to settle. Imaging was performed with a 40× oil-immersion objective (NA 1.3) using a 488 nm laser for YOYO-I excitation. All measurements were conducted at room temperature.

RE-dependent DNA Nanostar droplet degradation

To directly visualize RE-mediated degradation of DNA nanostars, two complementary experiments were conducted: (i) time-resolved confocal imaging of DNAns droplets (2 μM) before and after RE addition, and (ii) gel electrophoresis of DNAns solutions (0.4 μM) incubated with and without REs (the latter replaced by enzyme diluent buffer in control samples). DNAns Design A was used in both cases, with sample preparation protocols described in Sections III (positive control) and V.

Figure S2 shows the evolution of the average droplet size, $\langle S(t) \rangle$, over time in the absence of restriction enzymes (negative control). Although no enzymatic activity is expected under these conditions, the droplets appear to shrink by approximately 30% of their initial size after 30 minutes. This apparent decrease is attributed to photobleaching of the fluorescent signal during confocal imaging. As the fluorescence intensity gradually diminishes, the fixed-intensity threshold used in the image analysis algorithm underestimates droplet boundaries, leading to an apparent (but not physical) reduction in droplet size.

Consistent with previous findings[13], DNAns droplets shrank over time in the presence of REs due to DNA cleavage (Figure S3**(a)**, left). To quantify this process while correcting for photobleaching (Figure S2), the average droplet size in the presence of REs, $\langle \overline{S}(t) \rangle$, was measured at various time points and normalized by the size in the negative control, $\langle S(t) \rangle$. As shown in Figure S3**(a)** (right), $\langle \overline{S}(t) \rangle$ varied with enzyme type, revealing enzyme-dependent degradation kinetics: DNaseI (non-specific endonuclease) induced the fastest degradation, followed by EcoRI (two restriction sites per DNAns) and BtgI (one restriction site). All reactions used 1 U of enzyme per 0.38 μg of DNA, corresponding to an enzyme-to-DNA ratio of 2.6 U/μg.

To further confirm DNAns cleavage, the resulting fragments were analyzed by gel electrophoresis (Figure S3**(b)**). In these experiments, 1 μg of DNAns was digested. The NC displayed a single band corresponding to intact nanostars, whereas RE-treated samples exhibited distinct digestion patterns: DNaseI (2 U/μg) fully degraded the DNA, leaving no visible bands; EcoRI (20 U/μg) generated two fragments of smaller size (highlighted in green); and BtgI (10 U/μg) produced a single shifted fragment (highlighted in red).

These results confirm that DNAns within the phase-separation region undergo enzyme-specific degradation characterized by distinct kinetics and cleavage patterns depending on the restriction enzyme used.

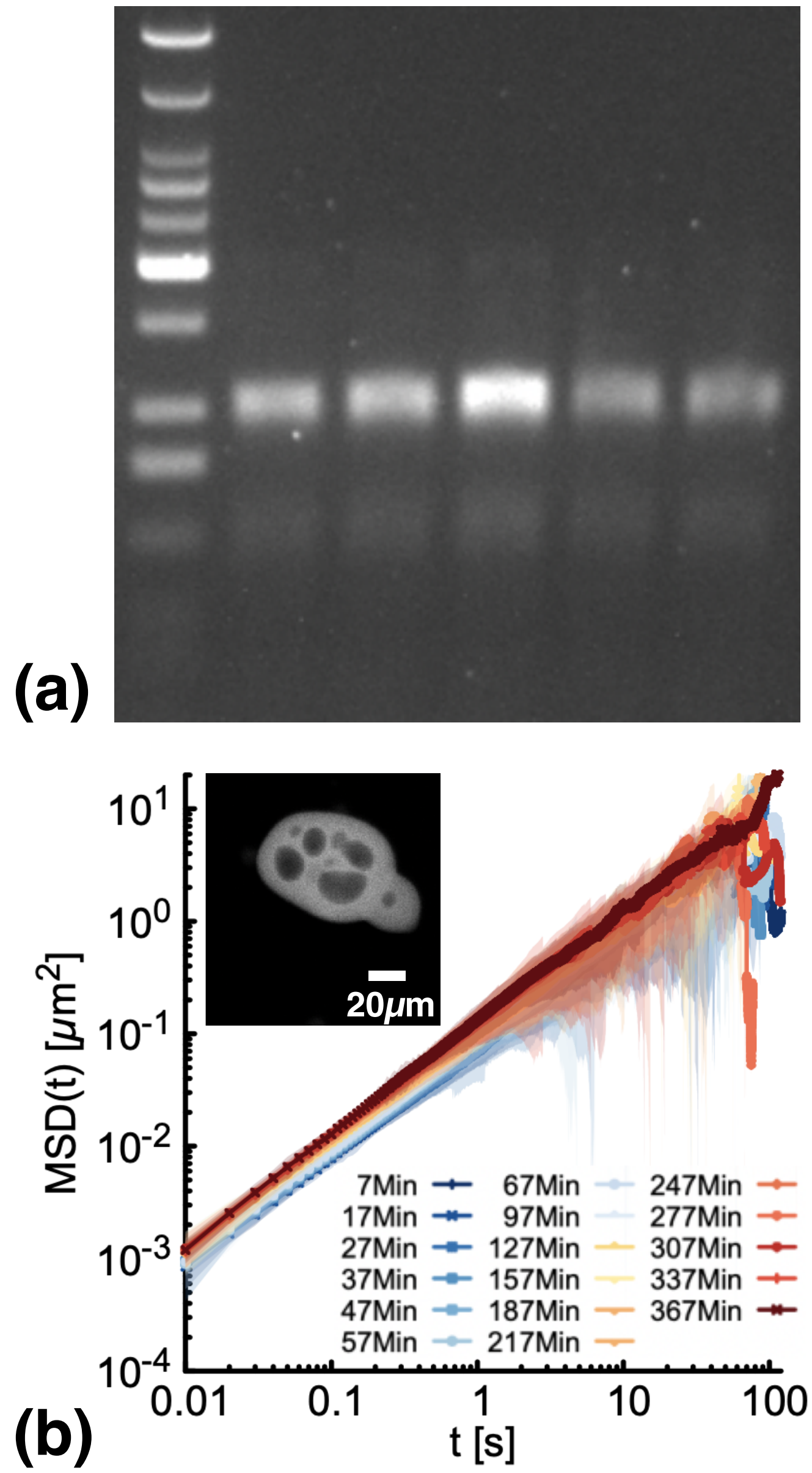

Fig. S4 Negative control for Design A samples. (a) Gel electrophoresis of the negative control (no enzyme added) for DNAns gels (Design A, 250 μM). No degradation is observed under DNaseI storage buffer after incubation at 35 °C for 0, 1, 2, 4, and 6 h. **(b)** Time-resolved microrheology of the same sample. The MSD(t) exhibits a predominantly liquid-like response with a slight upward shift over time (see also Figure **2(c)**). Inset: Confocal image showing the effect of glycerol on DNAns, with pore formation visible in droplets at 2 μM (scale bar: 20 μm).

VI Effect of glycerol on the rheology of DNA nanostars

To establish a baseline, the system was first characterized in the absence of enzyme (negative control). This ensured that any fragments or rheological changes observed in our experiments could be attributed solely to enzymatic activity. For gel electrophore-

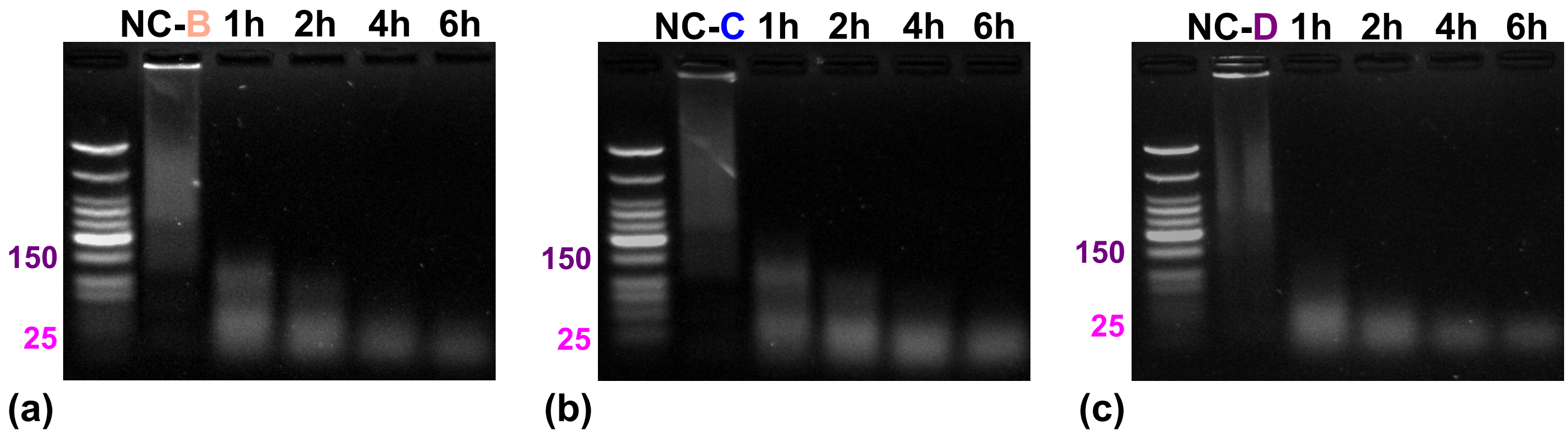

Fig. S5 DNase I degradation of different DNAns designs. Gel electrophoresis showing the degradation of DNAns hydrogels by DNase I over time (at 0.02 U/μg and 35 °C). Gel images display DNA digestion at 1, 2, 4, and 6 h. The 150 bp violet band corresponds to intact DNAns, while the 25 bp magenta band indicates smaller fragments resulting from enzymatic degradation. Data are shown for Designs B **(a)**, C **(b)**, and D **(c)**.

sis, a master solution of Design A DNAns (250 μM, $\sim$120 μg/μL) was prepared, in which the enzyme was replaced with its corresponding diluent buffer (1 mM Tris, 0.2 mM $CaCl_2$, 5% glycerol, pH 7.6 at 25 °C). The solution was incubated at 35 °C and aliquots were taken at specific time points. After six hours, a single band remained visible on the gel (Figure S4**(a)**), confirming that the nanostars remained intact.

Microrheology measurements supported this finding (Figure S4**(b)**). Under identical conditions, 200 nm tracer particles were tracked every 10 min for the first hour and every 30 min thereafter, up to six hours. Surprisingly, the samples exhibited predominantly liquid-like behavior, rather than the viscoelastic response reported previously[6]. In addition, a slight upward shift in the mean squared displacement over time was observed, indicating a gradual reduction in viscosity (see also Fig. **2(c)** of the main text). Although the molecular origin of this behaviour was not investigated directly, one possible explanation is the presence of 5% glycerol in the enzyme buffer. Glycerol has been reported to compete with water for hydrogen-bonding interactions and to alter DNA conformation in aqueous solutions[15]. Consistent with this interpretation, confocal imaging of DNAns droplets reveals pore formation following glycerol addition (Fig. S4(b), inset), suggesting that glycerol also modifies the structure of condensed DNA nanostar assemblies.

VII Non-specific enzyme DNase I digests all DNAns gel designs

In the main text, we demonstrated that DNase I efficiently digests gels composed of Design A DNAns. Here, we confirm that DNase I exhibits comparable efficiency in digesting DNAns solutions across the other three designs (B–D), as illustrated in Figure S5. For all three designs, digestion was performed at 35 °C over four time points (1, 2, 4, and 6 h) using the same enzyme-to-DNA ratio of 0.02 U/μg, where μg refers to the total DNA mass of nanostars plus linker.

As shown in Figure S5, the DNA in the negative controls for Designs B–D remains trapped in the well, likely due to the presence of longer sticky ends (8 nt versus 6 nt in Design A) and the linker, which promote the formation of more stable and larger structures even at low concentrations[16,17]. Upon DNase I addition, smaller DNA fragments appear in the gel, indicating complete digestion of the samples.

VIII ELISA

Based on the estimated pore size of the design D hydrogel, immunoglobulin G (IgG) antibodies were identified as compatible for capture and retention in DNAns hydrogels. IgG antibodies have approximate dimensions of 14.5 nm $\times$ 8.5 nm $\times$ 4.0 nm[18]. The antibody used in this study was Bevacizumab (Bio-Rad, MCA6089), a humanized monoclonal IgG that inhibits vascular endothelial growth factor (VEGF) and is used clinically for cancer therapy.

Hydrogel preparation. Two stock solutions were prepared: one containing only design D DNAns and one containing only linker (28 nt). Antibody was gently mixed with the linker solution at room temperature to achieve final concentrations of 1.68 mM linker and 0.114 μg/μL antibody. This mixture was then combined with the DNAns solution to form hydrogels with final concentrations of 0.33 mM DNAns, 0.5 mM linker, and 0.034 μg/μL antibody. To facilitate handling, the DNAns solution was heated to 45 °C for 5 minutes prior to mixing. Aliquots of 30μL hydrogel were placed at the bottom of 1.5 mL microfuge tubes. Negative control hydrogels (10 μL) were prepared using the same method but without antibody. Nine replicates of each hydrogel variant were prepared.

Hydrogel washing. Hydrogels were washed three times to remove unbound antibody and excess DNA. For each wash, three hydrogel volumes of nanostar buffer were gently added and removed without disturbing the hydrogel. Duplicate samples of the spent buffer from each wash were collected and stored at -20°C for later analysis.

Antibody retention assays. To assess retention, nineteen hydrogel volumes of nanostar buffer were added to each hydrogel and incubated at room temperature. At 0, 24, and 48 hours, duplicate samples of buffer were collected. For each collection, the buffer was carefully transferred to a fresh tube, vortexed, sampled in duplicate, and returned to the top of the hydrogel. Each

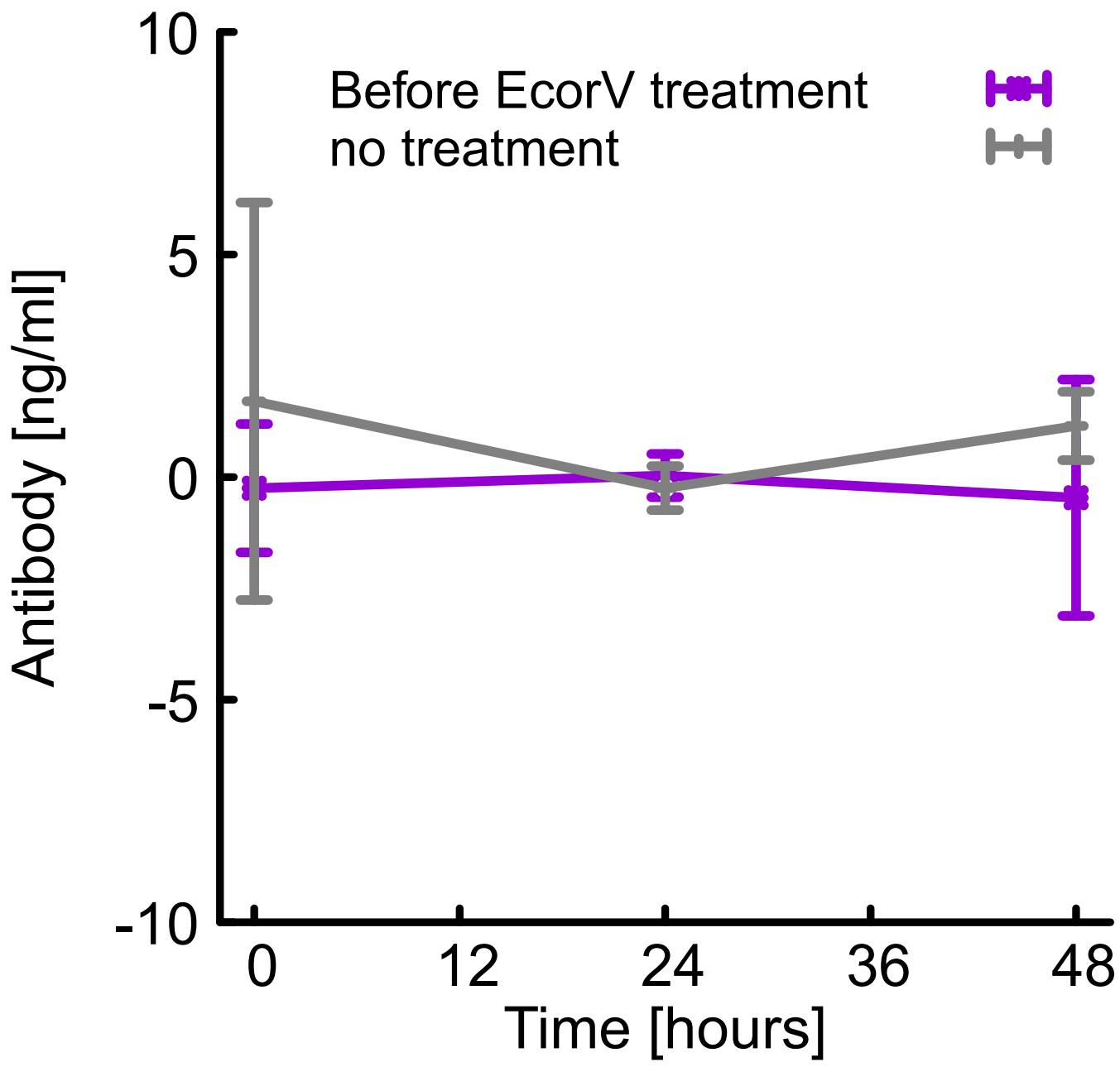

Fig. S6 Retention of IgG antibodies in design D DNA hydrogels in the absence of enzymatic activity. Antibody release profiles measured by ELISA for untreated hydrogels.

sample volume matched the hydrogel volume and was stored at $-20°$C until analysis. Results from ELISA experiments in Fig. S6 show that on average antibody levels never exceeded 2 ng/ml within the first 48 hours.

Controlled release assays. For controlled release experiments, hydrogels with and without antibody were treated with EcoRV-HF or no enzyme, all in triplicates. Prior to enzyme treatment, hydrogels were washed three times as described above. For treatment, nine hydrogel volumes of nuclease mixture were added to each hydrogel, and samples were incubated at room temperature. EcoRV-HF samples contained 0.68 U EcoRV-HF in 1× rCutSmart buffer, and no-enzyme controls were treated with water. Over a 73-hour time course, duplicate samples of equal volume to the hydrogel were collected as described above. Each sample was treated with 0.1 M EDTA to achieve a final concentration of 0.01 M for nuclease inactivation and stored at $-20°$C until analysis.

DNA integrity analysis. Enzymatic degradation of hydrogels was analyzed by agarose gel electrophoresis. A 3% agarose gel was prepared in TAE buffer, and 1 μL of samples collected during the nuclease treatment time course was loaded. Gels were run at 50 V for 90 minutes, corresponding to a field strength of 3.1 V/cm. Following electrophoresis, gels were post-stained with 1× SYBR Safe in 150 mL TAE and imaged using a Bio-Rad Molecular Imager® Gel Doc™ XR+ Imaging System with Image Lab software v6.1.

To directly assess hydrogel integrity, the intensity of the lowest–molecular-weight band, corresponding to DNA fragments released into solution, was quantified using ImageJ/Fiji. Prior to analysis, gel images were despeckled and background-subtracted to ensure uniform noise across both axes. Band intensities were manually selected using local intensity minima surrounding each band, and background signal was estimated from five randomly chosen regions lacking detectable bands and subtracted from each measurement. To enable quantitative comparison across different gels, the intensity of the lowest band in each sample lane was normalized by the intensity of the lowest band in the corresponding DNA ladder, which provides a consistent reference concentration across gels.

Notes and references

1 J. N. Zadeh, C. D. Steenberg, J. S. Bois, B. R. Wolfe, M. B. Pierce, A. R. Khan, R. M. Dirks and N. A. Pierce, *Journal of Computational Chemistry*, 2011, **32**, 170–173.

2 D. T. Nguyen and O. A. Saleh, *Soft Matter*, 2017, **13**, 5421–5427.

3 I. D. Stoev, T. Cao, A. Caciagli, J. Yu, C. Ness, R. Liu, R. Ghosh, T. O'Neill, D. Liu and E. Eiser, *Soft Matter*, 2020, **16**, 990–1001.

4 Y. Xing, E. Cheng, Y. Yang, P. Chen, T. Zhang, Y. Sun, Z. Yang and D. Liu, *Advanced Materials*, 2011, **23**, 1117–1121.

5 N. Conrad, T. Kennedy, D. K. Fygenson and O. A. Saleh, *Proceedings of the National Academy of Sciences*, 2019, **116**, 7238–7243.

6 G. Palombo, S. Weir, D. Michieletto and Y. A. Gutiérrez Fosado, *Nature materials*, 2025, **24**, 454–461.

7 N. E. Labs, *Optimizing Restriction Endonuclease Reactions.*, `https://www.neb.com/en/tools-and-resources/usage-guidelines/optimizing-restriction-endonuclease-reactions?srsltid=AfmBOoqTy4hZTfsh2YWp2jLhhf2DmqmkTF_zBMP_j7-K9FbgErYZ1QVH`.

8 P. Y. Lee, J. Costumbrado, C.-Y. Hsu and Y. H. Kim, *J Vis Exp.*, 2012, e3923.

9 C. R. Calladine and H. Drew, *Understanding DNA: The Molecule and How It Works.*, Academic Press, 1997.

10 A. D. Bates and A. Maxwell, *DNA Topology.*, Oxford University Press, USA, 2005.

11 J. R. Brody, E. S. Calhoun, E. Gallmeier, T. D. Creavalle and S. E. Kern, *BioTechniques*, 2004, **37**, 598–602.

12 A. D. Elliott, *Current Protocols in Cytometry*, 2020, **92**, e68.

13 O. A. Saleh, B. jin Jeon and T. Liedl, *Proceedings of the National Academy of Sciences*, 2020, **117**, 16160–16166.

14 B.-j. Jeon, D. T. Nguyen, G. R. Abraham, N. Conrad, D. K. Fygenson and O. A. Saleh, *Soft Matter*, 2018, **14**, 7009–7015.

15 D. Liang, L. Song, Z. Chen and B. Chu, *J Chromatogr A.*, 2001, **931**, 163–173.

16 Y. Sato, T. Sakamoto and M. Takinoue, *Science Advances*, 2020, **6**, eaba3471.

17 S. Agarwal, D. Osmanovic, M. A. Klocke and E. Franco, *ACS nano*, 2022, **16**, 11842–11851.

18 Y. H. Tan, M. Liu, B. Nolting, J. G. Go, J. Gervay-Hague and G.-y. Liu, *ACS Nano*, 2008, **2**, 2374–2384.
